# CryoEM structures reveal allosteric regulation of the catalytic activity of the multi-protein human MAT enzyme complexes

**DOI:** 10.1107/S2052252526005075

**Published:** 2026-06-22

**Authors:** Faisal T. Khaja, Reedhi Vara, Louie P. Aspinall, Ciara Merriman, Alana Maerivoet, Joshua B. R. White, Stephen P. Muench, S. Samar Hasnain, S. V. Antonyuk

**Affiliations:** a Molecular Biophysics Group, Department of Biochemistry, Cell and Systems Biology, Institute of Systems, Molecular and Integrative Biology University of Liverpool LiverpoolL69 7ZB United Kingdom; b Astbury Centre for Structural Molecular Biology University of Leeds LeedsLS2 9JT United Kingdom; c School of Biomedical Sciences, Faculty of Biological Sciences University of Leeds LeedsLS2 9JT United Kingdom; MRC Laboratory of Molecular Biology, United Kingdom

**Keywords:** methyl­ation, cancer, me­thio­nine cycle, SAMe, cell growth, allosteric regulation, drug discovery

## Abstract

S-Adenosyl me­thio­nine (SAMe), the biological methyl donor essential for sustaining the life of most complex organisms, is the second most widely used cofactor, after ATP, in biochemical reactions and is synthesized by the enzyme me­thio­nine adenosyl transferase (MAT) from ATP and me­thio­nine. MAT, also known as *S*-adenosylme­thio­nine synthetase, is found in almost every organism. SAMe is employed universally by different methyl­transferases that catalyze the methyl­ation of biomolecules such as nucleic acids, proteins and lipids. In plant cells SAMe produced by MAT enzymes controls the level of critical metabolites such as ethyl­ene, polyamines and biotin, and regulates essential cellular processes such as cell division and synthesis of cell wall, chloro­phyll and membrane. MAT enzyme complex MATα2β, comprising the catalytic unit MATα2 and the regulatory protein MATβ, is found in nearly all human tissues and is essential for providing the necessary SAMe flux for methyl­ation of DNA and various proteins including histones. The enzymatic activity of MATα2 is enhanced by several fold upon complexation with both variants of MATβ (βV1 and βV2). Using cryogenic electron microscopy, we determined the high-resolution resting-state structures of the MATα2_4_βV1_2_ and MATα2_4_βV2_2_ complexes, providing insights into the allosteric regulation of MAT catalytic activity, revealing how MATβV association could facilitate substrate binding, stabilize the transition state and promote product release to drive the catalytic cycle, and opening new possibilities for inhibitor binding.

## Abstract 2

High-resolution cryoEM structures of MAT enzyme complexes help to explain why MATα1 selectively forms a stable complex with MATβV1 but not with MATβV2, despite sharing high sequence and structural identity with MATα2.

## 1. Introduction

S-Adenosylme­thio­nine (SAMe), a universal methyl donor essential for cellular metabolism, is synthesized by me­thio­nine adenosyltransferase (MAT), also known as S-adenosylme­thio­nine synthase (Markham & Pajares, 2009 ▸; Kotb & Geller, 1993 ▸). This enzyme catalyses the conversion of me­thio­nine and ATP into SAMe, which serves as the principal methyl donor for a wide range of methyl­transferases (MT) (Markham & Pajares, 2009 ▸). As such, MAT enzymes are critical for maintaining cellular homeostasis by supporting DNA and protein methyl­ation, regulating mRNA m6A modification, and preserving molecular stability (Schlesier *et al.*, 2013 ▸; Strahl & Allis, 2000 ▸; Dominissini *et al.*, 2012 ▸; Grillo & Colombatto, 2008 ▸; Markham & Pajares, 2009 ▸). Beyond methyl­ation, SAMe functions as a key intermediate in the transsulfuration pathway, contributing to gluta­thione biosynthesis and redox balance, and plays an essential role in polyamine biosynthesis, thereby supporting cell growth, differentiation and apoptosis (Fernández-Ramos *et al.*, 2025 ▸) (Fig. S1).

MAT enzymes exist in multiple isoforms with distinct roles, and their expression and complex formation are tightly regulated by cellular conditions, including metabolic state, methyl­ation demand, oxidative stress and cell proliferation states (Ramani & Lu, 2017 ▸; Murray *et al.*, 2019 ▸). The tissue-specific distribution of MAT isozymes, together with the ability of cells to switch between isoforms, underscores the importance of regulatory mechanisms in controlling MAT function and cellular differentiation.

*MAT1A* encodes MATα1, which is predominantly expressed in the adult liver, where it maintains hepatic SAMe levels and supports liver function and regeneration (Mato *et al.*, 2008 ▸). Its downregulation is associated with liver diseases such as cirrhosis and hepatocellular carcinoma (HCC) (Barbier-Torres *et al.*, 2022 ▸; Llovet *et al.*, 2021 ▸; Murray *et al.*, 2019 ▸). In contrast, *MAT2A* encodes MATα2, which is expressed in extrahepatic tissues and proliferating cells, and is frequently upregulated in cancers to meet increased SAMe demand (Murray *et al.*, 2019 ▸). Two variants of MATβV, the regulatory subunit, are encoded by *MAT2B*, which on forming a complex with MATα2 enhance SAMe biosynthesis (Martínez-Chantar *et al.*, 2003 ▸, Murray *et al.*, 2019 ▸). *MAT2B* is overexpressed in aggressive cancers, such as triple-negative breast cancer (TNBC), where it contributes to tumour progression through increased proliferation and epigenetic alterations (Murray *et al.*, 2019 ▸; Peng *et al.*, 2015 ▸; Yang *et al.*, 2013 ▸; Xu *et al.*, 2019 ▸).

The MATβV isoforms share 94% sequence identity but differ in their N-terminal regions by 20 amino-acid residues. MATβV1 contains an extended N-terminus predicted to form a β-hairpin motif, whereas MATβV2 possesses a shorter, unstructured loop (Murray *et al.*, 2014 ▸). These structural differences are accompanied by distinct tissue-specific expression patterns: MATβV1 is predominantly expressed in the prostate, foetal liver, thyroid, lung, brain and adrenal gland, while MATβV2 is mostly found in skeletal muscle and the heart (Yang *et al.* 2008 ▸; Murray *et al.*, 2019 ▸). Both isoforms are present in the thymus and kidney (Peng *et al.*, 2015 ▸; Yang *et al.*, 2013 ▸). Dysregulation of MATβV isoforms is implicated in liver disease, cancer and neurological disorders, making them important therapeutic targets. The increasing levels of obesity have led to a high prevalence of non-alcoholic fatty liver disease (NAFLD), where higher levels of SAMe are present in serum consistent with an increased synthesis of SAMe in liver (Sáenz de Urturi *et al.*, 2022 ▸). Thus, MAT isozyme gene expression and subcellular localization have often been used as prognostic markers for liver, renal, breast and pancreatic carcinomas (Maldonado *et al.*, 2018 ▸).

MATα1 and MATα2 share 84% sequence identity with high structural similarity, and form a tetrameric assembly with four active sites, each composed of residues contributed by neighbouring subunits, reflecting the cooperative nature of catalysis (Kotb & Geller, 1993 ▸; Murray *et al.*, 2014 ▸, 2016 ▸; Panmanee *et al.*, 2020 ▸; González *et al.*, 2003 ▸). The enzymatic reaction proceeds via an S_N_2 mechanism in which l-me­thio­nine acts as a nucleophile to attack ATP to generate SAMe and intermediate tripolyphosphate (PPPi). This intermediate is subsequently hydrolysed at the β–γ bond, yielding inorganic phosphate (P_i_) and pyrophosphate (PP_i_) (Fig. S1) (O’Hagan & Schmidberger, 2010 ▸; Bailey *et al.*, 2021 ▸; Firestone & Schramm, 2017 ▸). The reaction products, SAMe, PP_i_ and P_i_, are then released from the enzyme. While MATα tetramers are catalytically active on their own, SAMe production is markedly enhanced upon complex formation with MATβV (Wan *et al.*, 2024 ▸; Panmanee *et al.*, 2019 ▸). Notably MATα2 activity increases by 450% and 250% in the presence of MATβV1 and MATβV2, respectively (Murray *et al.*, 2014 ▸). Mutation of Gln113 and Ser114 in the MATα2 gating loop severely impairs the enzymatic activity, which can only not be restored but enhanced compared with the wild type MATα2 alone upon interaction with MATβV isoforms, underscoring the importance of protein–protein interactions in regulating catalysis (Panmanee *et al.*, 2019 ▸, 2020 ▸; Murray *et al.*, 2016 ▸).

How such regulation and control occur through protein–protein interaction and complex formation remains largely unknown due to the scarcity of structural data on protein complexes. To date, structural information on MAT complexes is limited to a crystal structure of the MATα2–MATβV2 heterohexamer that we elucidated more than ten years ago (Murray *et al.*, 2014 ▸). The active site in this complex was occupied by a variety of ligands. The structure revealed an unexpected 4:2 stoichiometry and suggested that the binding of MATβV2 to MATα2 is mediated by its C-terminal motif. Crystallographic efforts have not succeeded either for a resting state (ligand-free) structure of the MATα2–MATβV2 complex or complexes with MATβV1.

Here, we employed cryogenic-sample electron microscopy (cryoEM; Henderson & Hasnain, 2023 ▸) to overcome limitations associated with crystallization and determined the apo structures of the heterohexameric MATα2_4_–MATβV1_2_ and MATα2_4_–MATβV2_2_ complexes. These structures provide the first resting-state snapshots of human MAT enzyme complexes, revealing key features of their architecture and offering new insights into isoform-specific allosteric regulation. Our findings advance the understanding of the first step of the me­thio­nine cycle and provide a structural framework for targeting MATβV-mediated metabolic dysregulation in disease.

## 2. Results

### 2.1. CryoEM structures of the MAT enzyme complexes (MATα2_4_βV1_2_ and MATα2_4_βV2_2_) in their resting states

**Figure 1:**
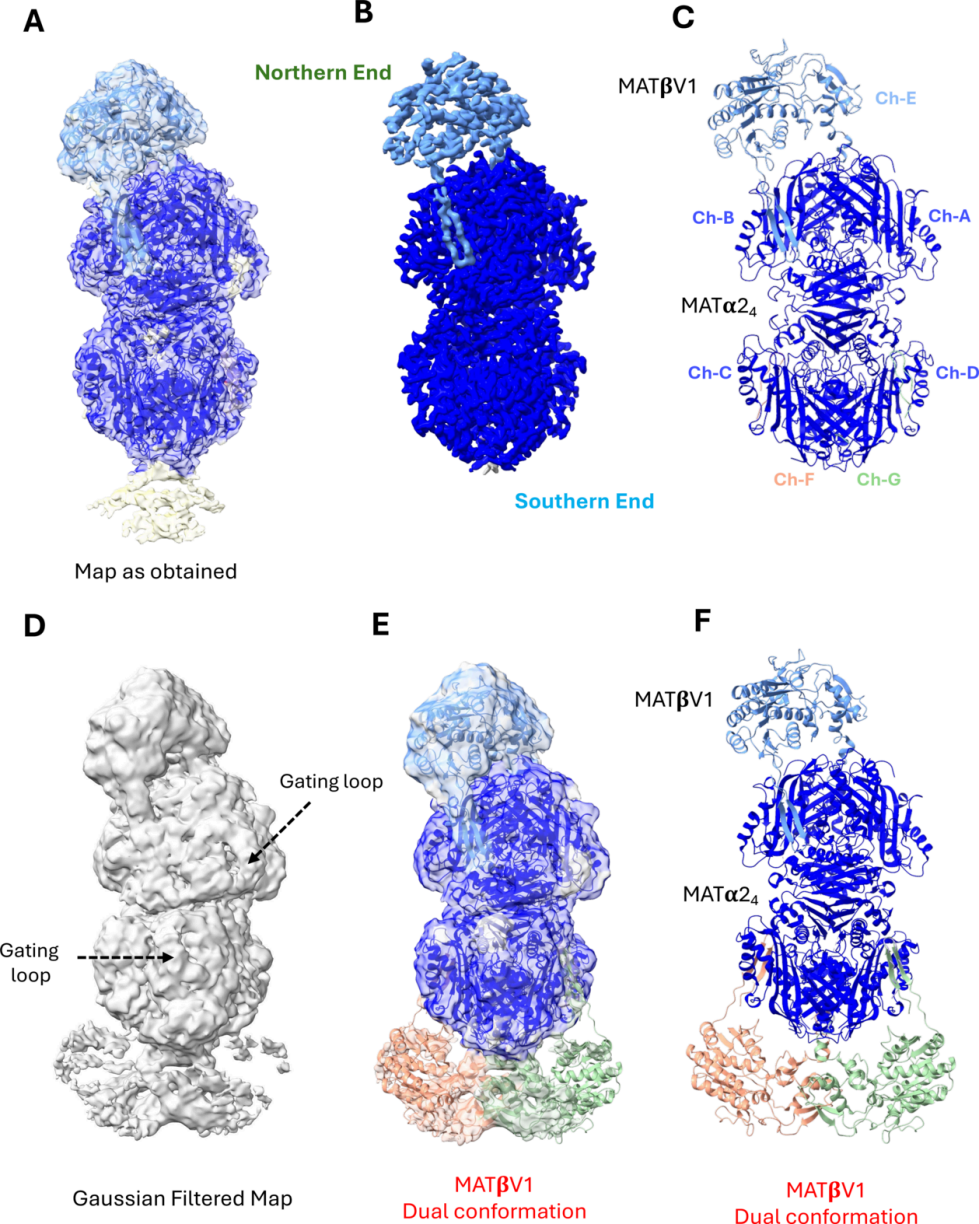
High-resolution cryoEM structure of the MATα2_4_βV1_2_ complex. (A) CryoEM Coulomb potential map of the MATα2_4_βV1_2_ complex determined by single-particle cryoEM at 2.6 Å resolution. (B) Composite cryoEM density map showing high-resolution density for the MATα2_4_ core (blue) and high-to-medium resolution density for MATβV1 at the northern end (cornflower blue). (C) Cartoon representation of the complex, highlighting the well defined MATα2_4_ core (blue) and a single, well resolved MATβV1 at the northern end (cornflower blue), alongside partially built MATβV1 subunits at the southern end adopting dual conformations shown in salmon and green. (D) Gaussian-filtered cryoEM map revealing the two conformations of MATβV1 at the southern end. (E) Cartoon model of MATα2_4_ with the northern MATβV1 (cornflower blue) and two MATβV1 subunits (salmon and light green) at the southern end rigid-body fitted into the Gaussian-filtered cryoEM map. (F) Overall view of the MATα2 tetramer (blue) interacting with MATβV1. MATβV1 at the northern end is shown in cornflower blue, while the southern end exhibiting a dual or wobbly conformation is represented in salmon and light green.

To shed light on how the activity of tetrameric MATα2 increases in the presence of MATβV1 and MATβV2, cryoEM structures of the MAT enzyme complexes MATα2_4_βV1_2_ and MATα2_4_βV2_2_ were determined in their resting states. The heterohexameric cryoEM structure of the MATα2_4_βV1_2_ complex is the first structure of this complex, consisting of a MATα2 tetramer flanked by a MATβV1 dimer, and was resolved at a resolution of 2.6 Å in the core region to approximately 6.0 Å in the peripheral regions (Fig. 1 ▸). The core, which represented a MATα2_4_ tetramer as well as one of the MATβV1 (referred to as the northern end) had well resolved EM density; however, MATβV1 bound at the other terminus (referred to as the southern end) displayed a high degree of conformational flexibility evident in the EM maps.

**Figure 2:**
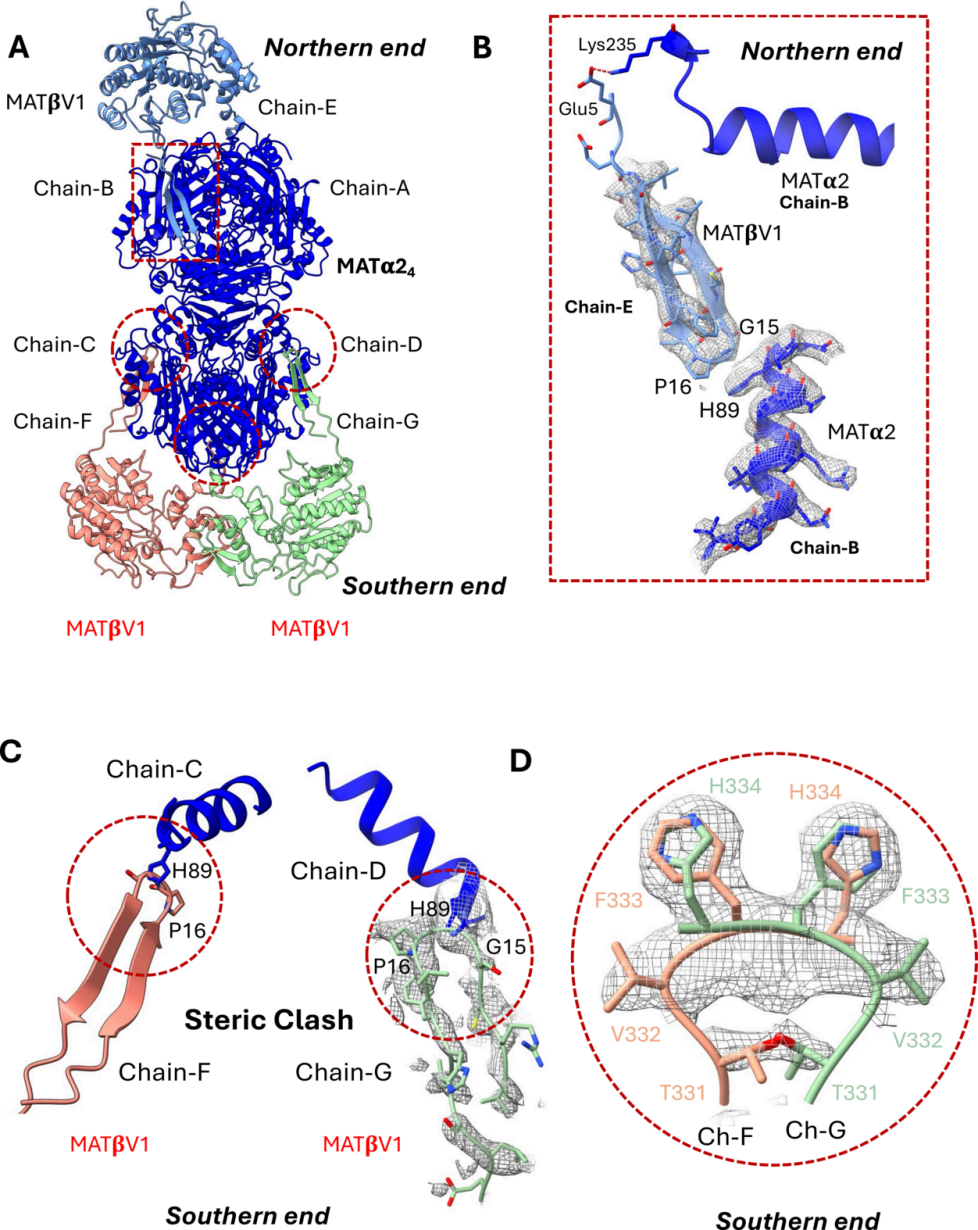
MATα2_2_ interaction with the MATβV1 N-terminus. (A) Cartoon representation of MATα2 tetramer (marine blue) interactions with MATβV1 (cornflower blue, salmon and light green). (B) Zoomed-in view of the MATα2 chain A interaction interface with the N-terminal hairpin of MATβV1, located within the MATα2 groove at the northern side; the ionic bond between Lys235 MATα2 and Glu5 MATβV1 is indicated by the dotted red line. His89 MATα2 in Chain A is in the conformation accommodating MATβV1 for the complex formation. (C) The steric clash of His89 MATα2 in Chain C and Chain D preventing full binding of the MATβV1 N-terminus (Coulomb density showing β-hairpin of one MATβV1 at the southern end modelled with 50% occupancy). (D) Coulomb density at the southern end interface showing two possible conformations of MATβV1 with equal probabilities of 50%.

**Figure 3:**
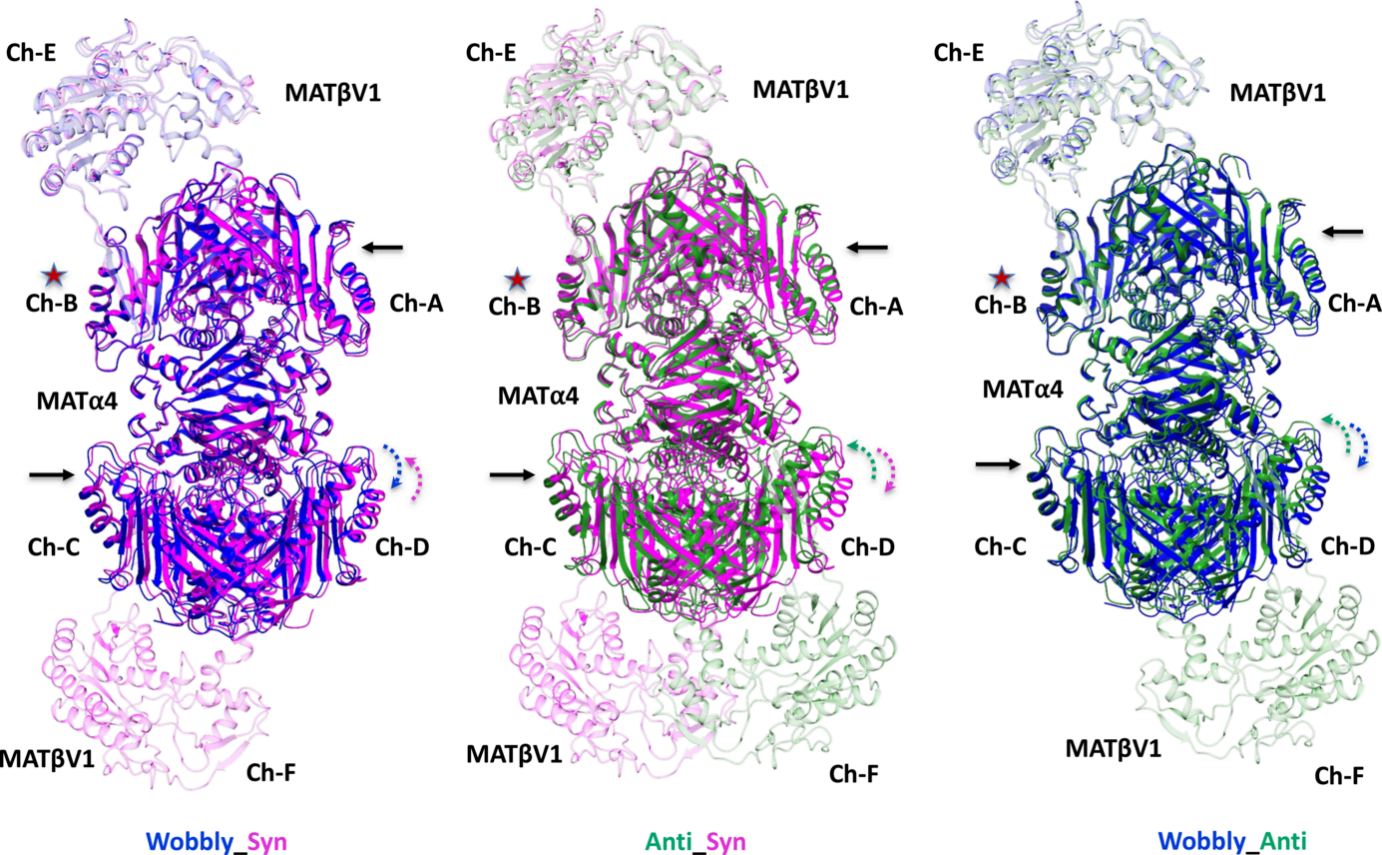
Chain-wise structural superposition reveals discrete conformational states and coordinated motion in the MATα2_4_βV1_2_ complex. Structural superposition of the three cryoEM classes of MATα2_4_βV12, wobbly, *syn* and *anti*, was performed using MATα2 Chain B as a reference (red star) to assess relative conformational changes across the complex. The three pairwise comparisons are shown: wobbly versus *syn* (left), *anti* versus *syn* (middle) and wobbly versus *anti* (right). MATα2 protomers (Chains A–D) and MATβV1 subunits (Chains E and F) are labelled. In all comparisons, the MATβV1 subunit at the northern end (Chain E) and the adjacent MATα2 protomers (Chains A and B) superimpose closely, indicating a rigid, well anchored interface. In contrast, the MATβV1 subunit at the southern end (Chain F) exhibits pronounced positional variability, accompanied by subtle but consistent shifts in the neighbouring MATα2 protomers (Chains C and D), highlighting propagation of motion into the catalytic core.

The explanation for this rotational flexibility or wobbly conformation near the southern end is indeed offered by the cryoEM structure of the MATα2_4_βV1_2_ complex, where the N-terminal of MATβV1 adopts a β-hairpin motif that folds back and lodges into a hydro­phobic pocket on the surface of one MATα_2_ monomer (Chain B) (Fig. 2 ▸). Several key intermolecular interactions, including ionic bonds, hydrogen bonding and cation–π interactions, are observed between the N-terminal of MATβV1 and the surface of MATα2 Chain A, increasing its buried surface area by approximately 50%. We found that a key hydro­phobic interaction between MATβV1-Gly15 and MATα2-His89 in one monomer (Chain B) holds MATβV1 at one end, thereby preventing its association with the adjacent monomer (Chain A). Structural superposition of MATβV1 onto the remaining two MATα2 monomers (Chains C and D) further reveals pronounced steric clashes between MATβV1-Gly15 and MATα2-His89, which preclude N-terminal engagement at the opposite monomeric interfaces (Fig. 2 ▸). Consequently, at the opposite southern end, MATβV1 is anchored solely through its C-terminal residues Val321-Phe322-His323, while the symmetric architecture of the MATα2 dimeric interface allows this subunit to wobble on either side (see *Methods*: *Model building*[Sec sec4.5]). However, if transiently stabilized by weaker interactions, the complex may adopt either a *syn* or *anti* conformation, depending on which side of the MATα2 dimer interface it engages. In both orientations, the MATβV1 tail is stabilized by the interactions involving identical residues of the MATα2 dimeric interface. This rotational flexibility was evident during data processing, where in addition to the predominant high-resolution ‘wobbly’ conformation a small fraction of MATα2_4_βV1_2_ complexes were also observed in both *syn* and *anti* conformational states, with the peripheral MATβV1 subunits in the southern end either aligned on the same side or on opposite sides of the MATα2_4_ core (Fig. S2). Given the dynamic nature of the complex and the conformational flexibility suggested by these cryoEM maps, 3D variability analysis (3DVA) in *CryoSPARC* was applied to explore both continuous and discrete motions across the particle populations. This approach enabled the identification of correlated domain movements and alternative structural states within each class, providing insight into the range of conformations adopted by the MATα2_4_βV1_2_ complex in solution (Supplementary movies 1–5). To further examine whether the observed heterogeneity reflects coordinated motions within the complex, we performed chain-wise structural superposition using MATα Chain B as a reference to compare the relative positioning of protomers across these classes (Fig. 3 ▸). This analysis revealed coordinated movement across MATα2 protomers, indicating that motions at the peripheral regulatory subunits are coupled to rearrangements within the catalytic core.

Notably, the amplitude of motion is asymmetric, and the largest conformational difference is observed between the *syn* and *anti* states, where the MATβV1 subunit at the southern interface undergoes a pronounced side-to-side reorientation across the MATα2 dimer interface. In contrast, the wobbly class represents a continuum of intermediate conformations that bridge these two extremes, resulting in an apparent reduction in displacement due to ensemble averaging. This indicates that the *syn* and *anti* conformations define the endpoints of a continuous rotational motion. *AlphaFold3* modelling is also consistent with these dual orientations of MATα2_4_βV1_2_ with nearly equal probabilities (Fig. S3).

An important ionic interaction may exist between MATβV1-Glu5 and MATα2-Lys235. Lys235 is part of a critical secondary structure that includes Phe250, a residue identified as important for substrate binding and product release during catalysis (Fig. 2 ▸B). Interestingly, the position of this secondary structure, and consequently that of Phe250, shifts during different stages of catalysis, highlighting the significance of this ionic interaction, which could play a role in allosteric regulation. Also, a common structural feature of the MAT enzyme complex is the ‘gating loop’ (amino acids 113–131) that flanks the active site. When the active site is occupied, this loop adopts a helical closed conformation, forming a gate over the catalytic pocket; in contrast, when the site is unoccupied, the loop becomes disordered or remains in an open conformation. The position of the gating loops in the MATα2_4_βV1_2_ complex is well defined at low threshold, with all four active sites of the MATα2 dimeric interface existing in an open conformation state consistent with the absence of any ligand/substrate bound to the active site (Fig. S4).

**Figure 4:**
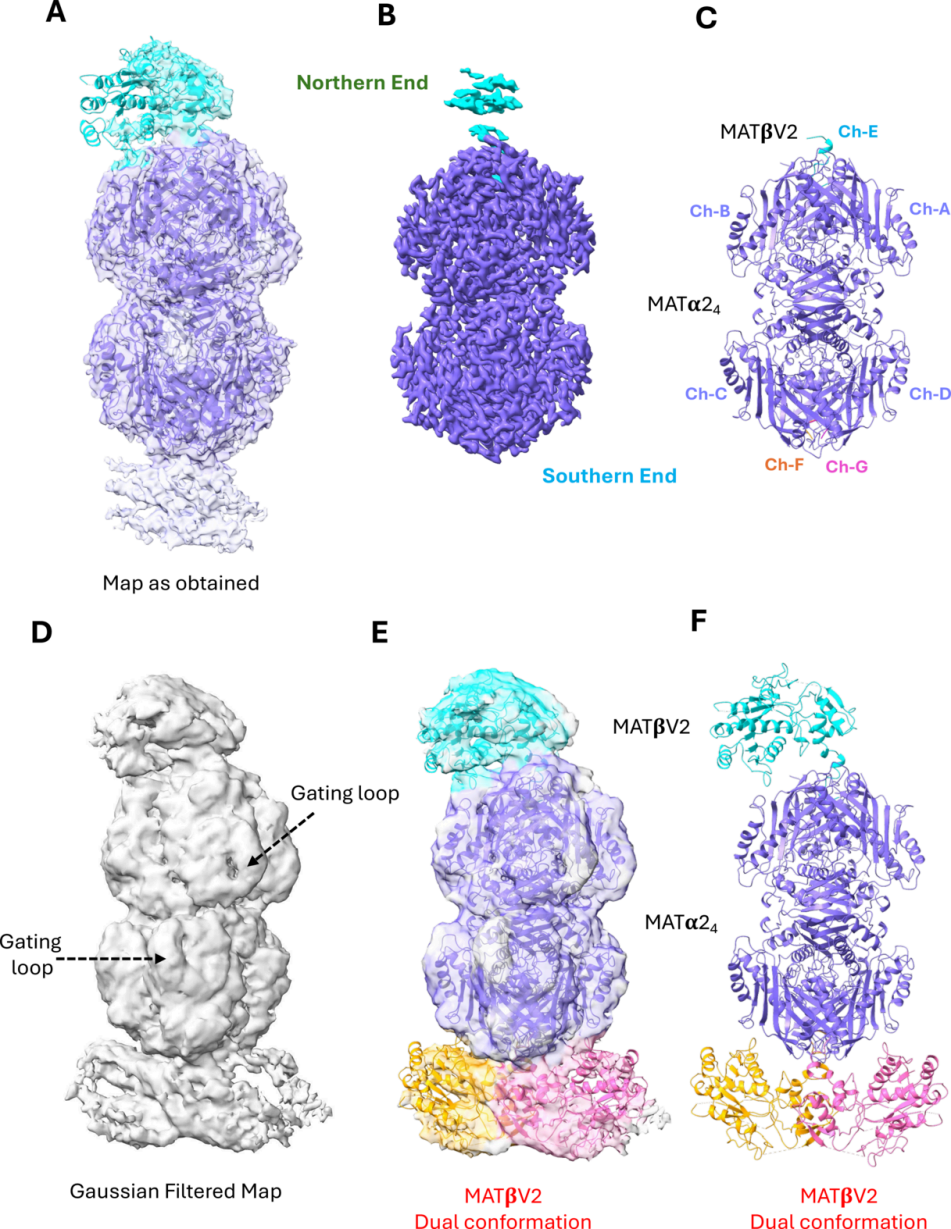
High-resolution cryoEM structure of the MATα2_4_βV2_2_ complex. (A) CryoEM Coulomb potential map of the MATα2_4_βV2_2_ complex determined by single-particle cryoEM at 2.6 Å resolution. (B) Composite cryoEM density, displaying high resolution for the MATα2_4_ core (medium slate blue) and lower resolution density for MATβV2 at the northern end (cyan). (C) Cartoon of the enzyme complex showing better-defined MATα2_4_ (medium slate blue) and single MATβV2 (cyan). (D) Gaussian-filtered cryoEM map showing a single conformation of MATβV2 at the northern end and dual or wobbly conformations at the southern end. (E) Cartoon representation of MATα2_4_, with a single MATβV2 at the northern end and two conformations of MATβV2 rigid-body fitted into a Gaussian-filtered cryoEM map. (F) Cartoon representation of the MATα2 tetramer (medium slate blue) interacting with MATβV2. MATβV2 at the northern end is shown in cyan, while the southern end exhibiting a dual conformation is represented in light orange and magenta.

To shed light on why the increase in activity of MATα2_4_ is lower when it associates with βV2 compared with βV1 (250 *vs* 450%) despite 94% sequence identity, we also determined the heterohexameric structure of the MATα2_4_βV2_2_ complex in the ligand-free resting state, at 2.6 Å, comprising a MATα2 tetramer flanked by a MATβV2 subunit on each end (Fig. 4 ▸). Like MATβV1, MATβV2 interacts with MATα2 by inserting its conserved C-terminal tail into the cleft at the MATα2 dimer interface, conferring rotational flexibility that enables engagement on both sides of the MATα2 active site (Fig. S5). Its N-terminal forms a disordered loop that could not be fully traced, but the MATβV2 at the northern end is partly stabilized in the 3D map, suggesting a weaker interaction with MATα2 compared with MATβV1. This explains the higher quality of the EM density near the northern end and why the dynamic conformation was predominantly observed at the southern end during data processing (Fig. S11). The greater stability of MATα2_4_βV1_2_ is consistent with its higher enzymatic activity compared with MATα2_4_βV2_2_, suggesting that these interactions play an important role in allosteric regulation and in enhancing the overall activity of the catalytic unit. In the MATα2_4_βV2_2_ complex, all gating loops were observed in the open conformation at low threshold (see *Methods*: *Model building*[Sec sec4.5]) (Fig. S4).

The *AlphaFold3* model of MATα2_4_βV2_2_ predicted MATβV_2_ in both *syn* and *anti* conformations, while the previously determined ligand-bound crystal structure captured it in the *anti* conformation only (PDB ID: 4ndn) (Fig. S3). This discrepancy underscores the importance of cryoEM data, which not only confirm the dual conformation of MATβV but also reveal conformational heterogeneity of the MAT enzyme complexes that might be missed in crystal structures due to the selective process imposed by crystallization conditions.

The EM density in the tetrameric core, corresponding to MATα2_4_, was well resolved, permitting a high confidence in model building. However, the peripheral region exhibited varying degrees of structural flexibility, with MATβV2 bound to one MATα2 dimer adopting a partially stable conformation at the northern end while the other, bound to the second MATα2 dimer at the southern end, displayed dynamic sampling even greater than that in MATα2_4_βV1_2_ with limited visibility in the final 3D reconstruction. Gaussian-filtered density maps clearly revealed a single conformation of MATβV2 at one end while a dual conformation at the other (Fig. 4 ▸). Similar to MATα2_4_βV1_2_, 3D variability analysis (3DVA) of the MATα2_4_βV2_2_ complex revealed coordinated domain rearrangements indicative of a cooperative breathing motion. These dynamics are clearly visualized in the gating loop, which alternates between ordered and disordered states and transitions between open and closed conformations in response to rotational movements of the MATβV2 subunits surrounding the active site (Supplementary movies 6–9).

### 2.2. Structural comparison of cryoEM apo MAT enzyme complexes with the ligand-bound MATα2_4_βV2_2_ crystal structure

The crystal structure of the MATα2_4_βV2_2_ holoenzyme complex was obtained in its post catalytic state where the products, such as SAMe, PPNP (an imidotriphosphate, a non-hydrolyzable triphosphate analogue) and metal ions, were trapped in the active site (Murray *et al.*, 2014 ▸). Superposition of the MATα2_4_βV1_2_ apo cryoEM structure onto the SAMe + PPNP-bound crystal structure of the MATα2_4_βV2_2_ complex revealed structural differences with an overall r.m.s.d. of ∼1.1 Å. Noticeable conformational differences were observed in the MATβV subunits, where MATβV1 appeared more stable than MATβV2 and is shifted downward towards the MATα2_4_ core by establishing additional contacts through its N-terminal β-hairpin motif. For example, subtle shifts were observed in MATβV2 positioning, and the MATα2 dimer interface cavity appeared more dilated in the ligand-bound complex compared with the apo structure. As a result, the three C-terminal residues of MATβV2 (Val321, Phe322, His323) in the ligand-complexed structure are repositioned deeper into the MATα2 dimeric interface, strengthening both ionic and hydro­phobic interactions (Fig. S6). MATβV2-His323 interacts with MATα2-Gly273 in Chain A and MATα2-Gly275 in Chain B, both of which are located within a critical loop containing key active site residues, including Lys285 and Lys265, that recognize the tripolyphosphate of ATP at the active site and play a direct role in catalysis. In the product-bound crystallographic structure, the distance between MATβV2-His323NE2 and MATα2-Gly273 (Chain A) is 2.8 Å, compared with 4.1 Å in the apo MATα2_4_βV1_2_ cryoEM structure, resulting in the loss of a hydrogen bond. A pronounced structural rearrangement was observed in the gating loop (residues 113–135), which was poorly resolved in the crystal structure for the open conformation but clearly visible in the cryoEM maps of MATα2_4_βV1_2_ and MATα2_4_βV2_2_ at low threshold. In the apo state, the distance between Phe250 (Chain B) and Ile117 (Chain A) is ∼24 Å, whereas in the SAMe-bound closed conformation, this distance decreases to ∼7.5 Å, reflecting a shift of ∼18 Å (Fig. S6). This movement is particularly evident in the position of Phe250, which participates in π–π stacking with the SAMe adenine ring in the product-bound structure.

Despite large changes in the SAMe binding region, particularly the position of Phe250, the positions of Glu23, His29, Lys181, Lys265, Lys285 and Asp291 which are involved in PPP binding (PPNP in the structure) are preserved in the product-bound and apo structures (Fig. S6). This suggests that the overall structural rearrangement occurs primarily in the SAMe binding/producing region, while the PPNP-binding region remains structurally conserved, explaining previous findings showing that SAMe production and ATP hydrolysis are independent of each other (Panmanee *et al.*, 2019 ▸). The dynamic motion in this region is further elucidated by comparing frames 1 and 20 of the volume series derived from 3D variability analysis (3DVA) of the final consensus map of the MATα2_4_βV2_2_ complex. The analysis reveals reversible remodelling of gating loops across all protomers, interconverting between open and closed states. These concerted transitions are coupled to rotational rearrangements of the MATβV2 subunits flanking the active site (Fig. S6; Supplementary movie 9).

These observations are also consistent with the comparison between the apo cryoEM and SAMe + PPNP-bound crystal structures of MATα2_4_βV2_2_, suggesting that the mode of substrate binding and product release is conserved between MATα2_4_βV1_2_ and MATα2_4_βV2_2_ and any differences in the rate of SAMe production are due to differences in protein–protein interaction with different regulatory MATβV subunits (βV1 and βV2) differentially facilitating substrate entry/binding and product release from MATα2 active sites. These structural changes upon complex formation in MATα2_4_βV2_2_ and MATα2_4_βV1_2_ are thus responsible for enhanced SAMe production compared with MATα2_4_ alone. Furthermore, greater stability of MATα2_4_βV1_2_ may help to provide a better configuration for catalysis and thus higher activity than MATα2_4_βV2_2_.

### 2.3. Allosteric regulation by MATβV

The binding of MATβV2 to MATα2 induces a series of conformational changes that propagate from the C-terminal cavity to the active site, influencing substrate binding, catalysis and product release. Previous studies have proposed that MATβV2 regulates the catalytic activity of MATα2 by altering its kinetic properties, specifically enhancing its affinity for l-me­thio­nine while reducing its sensitivity to SAMe inhibition (Wan *et al.*, 2024 ▸; Panmanee *et al.*, 2019 ▸; LeGros *et al.*, 2001 ▸). However, conflicting findings from a separate study suggest that MATβV2 binding primarily stabilizes MATα2 without significantly influencing its catalytic activity or SAMe inhibition (Bailey *et al.*, 2021 ▸).

In this study, we explored the structural basis of MATβV-mediated regulation of MATα2 by comparing the apo MATα2 (PDB ID: 6faj) and the product-bound (SAMe + PPNP) MATα2_4_βV2_2_ (PDB ID: 4ndn) crystal structures with the cryoEM MATα2_4_βV2_2_ and MATα2_4_βV1_2_ structures in their ligand-free (resting) states. Pairwise structural comparisons revealed that MATβV binding induces significant rearrangements in key catalytic residues near the active sites (Fig. S7). A central conformational shift involves Phe250, which, together with Ser247, Arg249 and Ile322, forms a critical pocket that stabilizes the adenine ring of SAMe during product formation. In the cryoEM structures, these residues adopt intermediate conformations, bridging the apo MATα2 (PDB ID: 6faj) and product-bound (MATα2_4_βV2_2_; PDB ID: 4ndn) structures. These intermediate rearrangements likely prime the active sites that can support more effective substrate binding, product formation and its release, highlighting a dynamic cycle of allosteric modulation contributing to efficient catalysis *i.e.* a higher level of SAMe formation compared with MATα2_4_ alone. Our findings demonstrate that the N-terminal loop and C-terminal tail of MATβV work in concert for repositioning Phe250, Arg249 and surrounding catalytic residues, effectively fine-tuning the active site geometry.

A markedly impaired SAMe synthesis has been demonstrated through mutational analysis of residues within the MATα1 active site, which is highly conserved and structurally similar to MATα2, (Fernández-Irigoyen *et al.*, 2010 ▸). Structural mapping of these mutations at Arg249, Ile252, Gly257, Asp258 and Ala259 resulted in defective me­thio­nine binding in MATα1. Arg249 is positioned between Phe250 and Ser248, within a flexible loop implicated in me­thio­nine entry and positioning within the active site.

Based on cryoEM maps of the MATα–MATβV complexes captured in multiple conformational states and 3D variable analysis, we propose that MATβV functions as an allosteric regulator, whose structural flexibility facilitates catalysis by modulating substrate entry, binding and product release, with each orientation (*syn* or *anti*) potentially favouring a distinct step of the reaction in response to different binding partners and varying metabolic or cellular conditions (Fig. S8).

Notably, the N-terminal β-hairpin of MATβV1, present in the MATα2_4_βV1_2_ complex which forms additional stabilizing interactions at the MATα2–MATβV1 interface, likely contributes to higher enzymatic activity compared to the complex with MATβV2.

## 3. Conclusion

Although it has been recognized that protein–protein interactions are fundamental to many control mechanisms *in vivo*, our understanding of how protein–protein interactions regulate enzyme activity, for example, has remained limited owing to a scarcity of high-resolution structures of complexes comprising the catalytic enzyme and regulatory protein (Keskin *et al.*, 2008 ▸; Jones & Thornton, 1996 ▸; Garner & Janda, 2011 ▸; Luck *et al.*, 2020 ▸). This is also the case for me­thio­nine adenosyl transferase (MAT) enzyme complexes that catalyse the formation of SAMe by utilizing me­thio­nine and ATP. Higher levels of SAMe formation are achieved when the catalytic subunit MATα complexes with different isoforms of the regulatory subunit MATβ. The ligand-bound (with SAMe, PPNP and metal ions bound) crystallographic structure of the MATα2_4_βV2_2_ complexes (Murray *et al.*, 2014 ▸) together with the cryoEM structures of MATα2_4_βV1_2_ and MATα2_4_βV2_2_ complexes, resolved here in their apo forms, provide a structural roadmap of the enzyme’s catalytic cycle, allowing us to shed light on the structural elements in MATβV that are likely to be important for complex formation and allosteric regulation.

**Figure 5:**
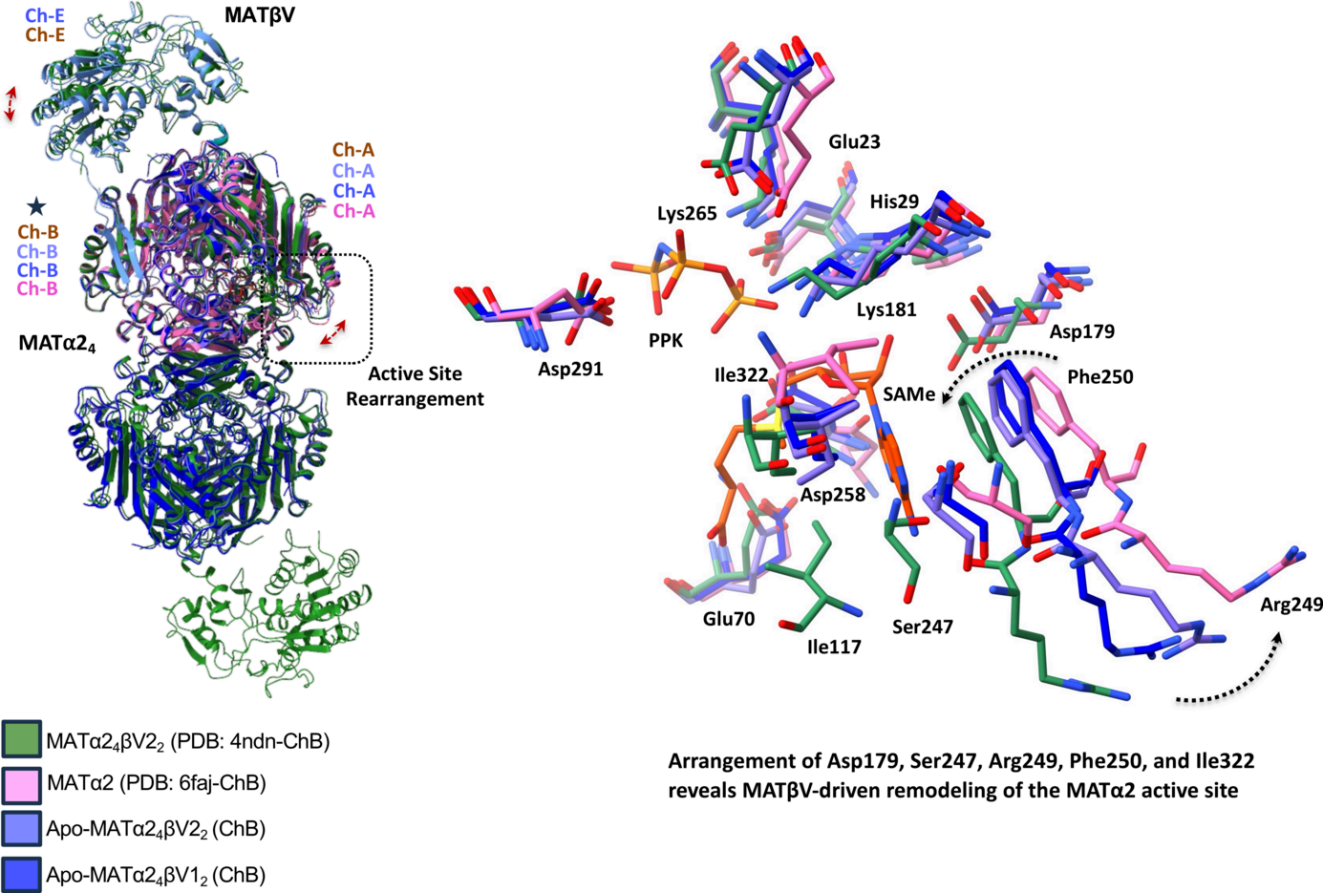
Key structural insights into control, regulation and allosteric mechanism of MAT enzyme complexes. Chain-specific structural superposition of the MATα2 protomer in apo MATα2_4_ (PDB ID: 6faj, Chain B), the SAMe + PPNP-bound MATα2_4_βV2_2_ complex (PDB ID: 4ndn, Chain B), and the resting-state cryoEM structures of MATα2_4_βV2_2_ (Chain B) and MATα2_4_βV1_2_ (Chain B) illustrates allosteric conformational changes near the active-site pocket upon MATβV binding. The right inset presents an expanded stick view of the active-site pocket, highlighting the spatial orientation of key residues and showing how MATβV modulates the architecture of the MATα2 active site. The MATα2 protomers used as references for chain-specific structural superposition are indicated with asterisks, and all structures are colour-coded as indicated.

Structural comparison between ligand-bound crystal structures and the cryoEM apo structure of the MATα2_4_βV2_2_ complex reveals a pronounced repositioning of MATβV2, accompanied by dilation of the cavity at the MATα2 dimer interface, consistent with observations from 3D variability analysis for both heterocomplexes. These interactions promote structural rearrangements that affect the gating-loop conformation that could reposition Arg249 and Phe250, key residues for controlling substrate access and product release (Fig. 5 ▸). These comparisons also allow us to conclude that the greater stability of MATα2–MATβV1 compared with MATα2–MATβV2 arises due to the presence of the N-terminal β-hairpin, which interacts with a hydro­phobic patch on the MATα2 surface. MATβV2 lacks the N-terminal β-hairpin and relies solely on the C-terminal tail for binding to MATα2, resulting in a less stable complex.

The stability of the complex correlates well with the enhancement of enzymatic activity of MATα2, which is increased substantially but to varying levels when complexed with MATβV1 and MATβV2, respectively (Murray *et al.*, 2014 ▸). The stable nature of these complexes, admittedly of varying degree of stability, and their direct impact on the catalytic activity suggest that these complexes represent obligatory associations of MATα2 and βV1/βV2 with profound functional impact and as such they are also likely to be more relevant for drug development and *in vivo* testing. The obligatory associated complexes differ from the transient encounter complexes, such as the electron transfer complexes, where the heterocomplex interfaces are generally less extensive and the partner proteins have weaker associations (Keskin *et al.*, 2008 ▸). Unlike the obligatory enzymatic complexes, the interaction between partner proteins in an electron transfer complex, such as cytochrome *c* and cytochrome *c* oxidase, is weak and dynamic, enabling frequent on-off exchange of partner proteins (Sakamoto *et al.*, 2011 ▸). The presence of multiple conformational states (wobbly, *syn* and *anti*) suggests that the β-hairpin insertion in MATβV1 not only enhances overall complex stability but also restricts the extent of conformational sampling, thereby modulating long-range allosteric communication between distal regulatory sites and the active site.

The differential activity of MATα2–MATβV1 and MATα2–MATβV2 suggests distinct regulatory roles of MATβV in SAMe production, potentially adapting to specific metabolic demands and cellular conditions. The stability of the MATα2–MATβV1 complex may provide a robust platform for sustained enzymatic activity, ensuring a steady supply of SAMe for methyl­ation reactions. This is particularly important in rapidly dividing cells with high metabolic demands, such as cancer cells, embryonic stem cells and the immune cells during activation. In contrast, the transient nature of the MATα2–MATβV2 complex may limit its ability to sustain high levels of SAMe production over extended periods, making it less suitable for tissues with constant high metabolic demands. This tiered regulation – with MATβV1/V2 serving as metabolic rheostats – ensures precise SAMe allocation, while its dysregulation creates therapeutic vulnerabilities. For example, the liver’s *MAT1A*-to-*MAT2A* switch in hepatocellular carcinoma (HCC) and the overexpression of MATβV1 in aggressive cancers such as triple-negative breast cancer (TNBC) exemplify how shifting complex composition mirrors pathological SAMe requirement (Murray *et al.*, 2019 ▸; Xu *et al.*, 2019 ▸).

The cryoEM structures in this study also help to explain why MATα1 selectively forms a stable complex *in vitro* with MATβV1 but not with MATβV2, despite sharing high sequence and structural identity with MATα2 (Panmanee *et al.*, 2020 ▸). Structural studies of hetero complexes of MATα2 show that stable complex formation requires two key interaction modules in MATβV: the C-terminal tail, which inserts into the MATα dimeric interface, and the N-terminal β-hairpin, which engages a hydro­phobic surface patch. Because MATβV1 contains both elements, whereas MATβV2 lacks the β-hairpin, MATβV1 consistently binds more strongly to MATα subunits and enhances catalytic output to a greater extent. *In silico* modelling of MATα1–MATβV complexes further revealed that although MATβV2 can fit into the MATα1 interface without steric clashes, variant residues in MATα1 – such as the substitutions of Arg192→Asn and Ile196→Leu – disrupt critical ionic and hydro­phobic interactions that normally stabilize the MATα2–MATβV2 complex (Fig. S10). Together, these findings suggest that subtle sequence and structural divergence among MATβ isoforms modulates their interaction landscape, thereby fine-tuning MATα activity under specific cellular conditions. Despite substantial conformational heterogeneity, these complexes remain stable, indicating that MATα2 forms obligatory functional associations with βV1 and βV2. These complexes, therefore, represent the biologically relevant state of the enzyme and are appropriate targets for drug development and *in vivo* studies, particularly in cancer, where MATα2 and the two major *MAT2B* variants (MATβV1 and MATβV2) are overexpressed, conferring growth advantage.

## 4. Materials and methods

### 4.1. Protein expression and purification of MATα1, MATα2, MATβV1 and MATβV2

All the MAT enzyme constructs (MATα1, MATα2, MATβV1 and MATβV2) were produced in *E. coli* BL21 (DE3 strain) and grown in lysogeny broth (LB) with 50 mg ml^−1^ of kanamycin for antibiotics selection, as described in detail before (Panmanee *et al.*, 2019 ▸). Cells were lysed using a hydraulic press cell disruptor at 28 000 p.s.i and cell debris was then removed by centrifuging at 20 000g for 45 minutes at 4°C. The proteins were eluted with elution buffer [25m*M* HEPES pH 7.5, 500m*M* NaCl, 250m*M* imidazole, 5%(*v*/*v*) glycerol and 1m*M* DTT]. The fractions containing the MATα1 or MATα2 were pooled and incubated overnight with tobacco etch virus (TEV) protease in a ratio of 100:1 (protein:protease) at 4°C in dialysis buffer [25m*M* HEPES pH 7.5, 500m*M* NaCl, 5%(*v*/*v*) glycerol and 1m*M* DTT]. The fractions containing MATβV1 or MATβV2 were incubated with Sentrin Specific Protease 2 (SENP-2) and dialyzed overnight. Final proteins were eluted in SEC buffer [25m*M* HEPES pH 7.5, 500m*M* NaCl, 5%(*v*/*v*) glycerol and 0.5m*M* TCEP], concentrated, and stored at −80°C.

### 4.2. Formation of the MATα2_4_βV2_2_ and the MATα2_4_βV1_2_ complexes

The complex formation protocol was performed as previously reported (Murray *et al.*, 2014 ▸). Briefly, MATα2 was incubated with MATβV2 or MATβV1 in the buffer (50m*M* HEPES pH 7.5, 150m*M* NaCl and 1m*M* DTT) for 2 h at 4°C. Samples were loaded onto a Superdex 200 10/300 gel filtration column and the complexes were eluted. Fractions containing each complex were pooled and concentrated to 5 mg ml^−1^ and quantified using the Bradford assay.

### 4.3. CryoEM grid preparation and data collection

MATα2βV1 and MATα2βV2 were diluted from 5 mg ml^−1^ to optimal concentrations of 0.125 mg ml^−1^ and 0.25 mg ml^−1^, respectively. Quantifoil R 1.2/1.3 300 mesh grids (Quantifoil^TM^) were glow discharged for 2 minutes at 12 mA, within a PELCO easiGLOW glow discharging device. Vitrification and grid formation was undertaken using the Vitrobot Mark IV vitrification device. 3.5 µl of sample was applied to the grids, with a wait time of 10 s before blotting, and with a blot time of 6 s and blot force of 1. Imaging was performed with a Titan Krios G2 microscope, operated at 300 kV with an X-FEG. Movies were collected in *EPU* software (version 3.8), using a Falcon 4i detector with a Selectris energy filter, at a pixel size of 0.74 Å (165 000 magnification) (Grollios *et al.*, 2024 ▸). For MATα2βV1 and MATα2βV2, 10 375 and 10 707 movies were collected in counting mode, using a total dose of 40 e^−^ Å^−2^ and dose rate of 7.25 e^−^ pixel^−1^ s^−1^ and 7.41 e^−^ pixel^−1^ s^−1^, respectively (Supplementary Table 1).

### 4.4. CryoEM data processing

The cryoEM data-processing workflows for the MATα2_4_βV2_2_ and MATα2_4_βV1_2_ datasets are illustrated in Supplementary Figs. 11–19. The datasets were processed using an integrated approach combining *CryoSPARC* (version 4.6.0) and *RELION* (version 4.0.2), with *crYOLO* for particle picking (Punjani *et al.*, 2017 ▸; Scheres, 2012 ▸; Wagner *et al.*, 2019 ▸). For the MATα2_4_βV2_2_ complex, 10 707 movies were motion corrected, followed by contrast transfer function (CTF) estimation using *CTFFIND-4.1* and 2D classification to select ∼4.5 million particles (Rohou & Grigorieff, 2015 ▸). These were used for *abinitio* model building and heterogeneous refinement. A major class displaying the MATα2 tetramer and partial MATβV2 density was subjected to 3D classification, yielding a well defined MATα2_4_βV2_2_ reconstruction comprising ∼135 219 particles (Class II) that was refined to 2.61 Å resolution, with focused local refinement improving the MATβV2 density near the northern end to ∼4.2 Å (see the supporting information).

Processing for the MATα2βV1 dataset followed a similar procedure, where 10 375 movie stacks were motion-corrected, followed by CTF estimation, 2D classification, *abinitio* model building and heterogenous refinement to select ∼1 million particles. These were used for *ab initio* model building and heterogeneous refinement. A major class displaying the MATα2 tetramer and clear MATβV1 density near the northern end, along with the dual conformation near the southern end, was subjected to 3D classification. This yielded a well resolved MATα2_4_βV1_2_ reconstruction comprising ∼122 584 particles (Class II) that was refined to 2.61 Å resolution. Subsequent focused local refinement improved the MATβV1 density at the northern end to ∼3.3 Å. A subset of ∼51 300 particles from Class IV, featuring MATβV1 subunits in *anti* conformation relative to the MATα2_4_ core, was refined to a resolution of 3.2 Å. Using an alternative focused 3D classification strategy, ∼95 000 particles (Class VI) corresponding to the MATα2_4_βV1_2_ complex in a *syn* conformation – where both MATβV1 subunits are aligned in the same direction – were identified and refined to a final resolution of 2.85 Å. 3D variability analysis in *CryoSPARC* was conducted on the final particle sets to characterize both discrete and continuous structural heterogeneity of the reconstructed volumes (see the supporting information) (Punjani *et al.*, 2017 ▸).

### 4.5. Model building

Model building for the resting state of MATα2_4_βV2_2_ was initiated by segmenting the high-resolution ligand-bound MATα2_4_βV2_2_ crystal structure (PDB ID: 4ndn) into MATα_2_ and MATβV_2_ subunits. The MATα2 subunits were rigid-body fitted into well resolved regions of the cryoEM density corresponding to the tetrameric core using *ChimeraX* (Meng *et al.*, 2023 ▸). All gating loops were observed in an open conformation at low threshold (Fig. S4), and model building was performed manually in *COOT* only for the gating loop in Chain D, while those in the remaining chains were left unmodelled (Emsley & Cowtan, 2004 ▸). The EM density at the northern end enabled modelling of only the C-terminal residues of MATβV2 (residues 311–323), whereas the N-terminal region and the remainder of the protein (residues 1–310) were fitted as a rigid body into the EM map and were not included in the deposited structure (PDB ID: 9qpo). Notably, the last four C-terminal residues of MATβV_2_ at the southern end (Thr320, Val321, Phe322 and His323) were modelled in two distinct conformations, each with 50% occupancy. Subsequent refinement was carried out in *Phenix* (Liebschner *et al.*, 2019 ▸). The remaining portions of the MATβV2 subunit near the southern end were rigid-body fitted into the low-threshold map in dual conformation at equal occupancies (50%) for structural interpretations (not included in the deposited structure), reflecting the conformational heterogeneity and wobbly nature of this region.

Model building for the MATα2_4_βV1_2_ resting state EM map followed a similar strategy, using segmentation of the ligand-bound MATα2_4_βV2_2_ crystal structure (PDB ID: 4ndn) into MATα2 and MATβV1 subunits, followed by rigid-body fitting into the cryoEM density using *ChimeraX* (Meng *et al.*, 2023 ▸). The gating loop was manually built in *COOT* for Chain B using low-threshold EM maps, while those in the remaining chains were left unmodelled. The MATβV subunit at the northern end was better resolved and fitted the EM density more clearly, allowing modelling of missing N-terminal residues in *COOT* (Emsley & Cowtan, 2004 ▸). Notably, at the southern end, the N-terminus of MATβV1 (Chain F and Chain G: residues 7–19) was built into weak EM densities on both sides of the MATα2 dimeric interface (Fig. 2 ▸C), showing steric clash and unstable interaction, while the C-terminal end residues (332–334) were modelled in two distinct conformations. Subsequent refinement was performed using the Servalcat *REFMAC5* controller implemented in the *CCP-EM* package (Burnley *et al.*, 2017 ▸). The remaining portions of each MATβV1 subunit near the southern end were rigid-body fitted into the low-threshold map at equal occupancies (50%) only for structural interpretations and were not included in the deposited structure (Figs. 1 ▸E and 4 ▸E), reflecting substantial flexibility and conformational heterogeneity in this region.

Model building of the MATα2_4_βV1_2_ complexes in the *syn* and *anti* conformations included the MATα2_4_ core and two MATβV1 subunits rigid-body fitted into their respective positions using *ChimeraX* and *iSOLDE*, followed by real-space refinement in *Phenix* (Meng *et al.*, 2023 ▸; Croll, 2018 ▸; Liebschner *et al.*, 2019 ▸). The final structures (PDB IDs: 30gd and 30gh) excluded gating loops, which could not be modelled owing to insufficient density. The stereochemical quality of all structures was assessed using *COOT* and *MolProbity*, and comprehensive model validation was performed using *Phenix* (Supplementary Tables 1 and 2) (Emsley & Cowtan, 2004 ▸; Davis *et al.*, 2007 ▸). Figures were generated using *PyMOL* (https://www.pymol.org) and *ChimeraX* (Meng *et al.*, 2023 ▸).

The MATα2 protomer used as a reference for chain-specific structural superposition is indicated with an asterisk in Fig. 5 ▸. Notably, the active site of MATα2 (Chain B) in the product-bound MATα2_4_βV2_2_ complex (PDB ID: 4ndn) is unoccupied; therefore, this protomer was selected as the reference for structural comparison with apo MATα2 (PDB: 6faj, Chain B) and the resting-state cryoEM structures of MATα2_4_βV2_2_ (Chain B) and MATα2_4_βV1_2_ (Chain B). MATβV adopts a similar conformation across these structures, enabling identification of structural changes and comparison of active-site residues in the ligand-bound MATα2_4_βV2_2_ complex (PDB ID: 4ndn, Chain A), apo MATα2 (PDB ID: 6faj, Chain A), and the cryoEM structures of MATα2_4_βV2_2_ and MATα2_4_βV1_2_ (Chain A).

## Supplementary Material

Supplementary experimental details, figures and tables, and captions for movies. DOI: 10.1107/S2052252526005075/hen5003sup10.pdf

Supplementary movie 1. DOI: 10.1107/S2052252526005075/hen5003sup1.mp4

Supplementary movie 2. DOI: 10.1107/S2052252526005075/hen5003sup2.mp4

Supplementary movie 3. DOI: 10.1107/S2052252526005075/hen5003sup3.mp4

Supplementary movie 4. DOI: 10.1107/S2052252526005075/hen5003sup4.mp4

Supplementary movie 5. DOI: 10.1107/S2052252526005075/hen5003sup5.mp4

Supplementary movie 6. DOI: 10.1107/S2052252526005075/hen5003sup6.mp4

Supplementary movie 7. DOI: 10.1107/S2052252526005075/hen5003sup7.mp4

Supplementary movie 8. DOI: 10.1107/S2052252526005075/hen5003sup8.mp4

Supplementary movie 9. DOI: 10.1107/S2052252526005075/hen5003sup9.mp4

PDB reference: MATα2_4_βV1_2_, wobbly, 9qpp

PDB reference: MATα2_4_βV2_2_, wobbly, 9qpo

PDB reference: MATα2_4_βV1_2_, *syn*, 30gd

PDB reference: MATα2_4_βV1_2_, *anti*, 30gh
