# Supplementary material for: CryoEM structures reveal allosteric regulation of the catalytic activity of the multi-protein human MAT enzyme complexes
Source: IUCrJ. 2026 Jun 22;13(Pt 4):373–84. doi: 10.1107/S2052252526005075 (PMC13324607; doi:10.1107/S2052252526005075)
Supplement: Supplementary file 1 [file m-13-00373-sup10.pdf]

IUCrJ

Volume 13 (2026)

Supporting information for article:

# CryoEM structures reveal allosteric regulation of the catalytic activity of the multi-protein human MAT enzyme complexes

Faisal Khaja, Reedhi Vara, Louie P. Aspinall, Ciara Merriman, Alana Maerivoet, Joshua B. R. White, Stephen P. Muench, S. Samar Hasnain and S. V. Antonyuk

# Supporting Information

# cryoEM structures reveal allosteric regulation of the catalytic activity of the multi-protein human MAT enzyme complexes

Faisal T. Khaja[1], Reedhi Vara[1], Louie P. Aspinall[2], Ciara Merriman[1], Alana Maerivoet[1], Joshua B.R. White[2], Stephen P. Muench[2,3]*, S Samar Hasnain[1]* and S. V. Antonyuk[1]*

[1]Department of Biochemistry, Cell and Systems Biology, Institute of Systems, Molecular and Integrative Biology, University of Liverpool, Liverpool, L69 7ZB, UK;

[2]Astbury Centre for Structural Molecular Biology, University of Leeds, Leeds, LS2 9JT, UK

[3]School of Biomedical Sciences, Faculty of Biological Sciences, University of Leeds, Leeds, LS2 9JT, UK;

## CryoEM data processing

The cryoEM data processing workflows for the $MAT2_4V2_2$ and $MAT2_4V1_2$ datasets are illustrated in **Supplementary Figure 11-19** respectively. The datasets were processed using an integrated approach combining CryoSPARC (version 4.6.0) and RELION (version 4.0.2) (Punjani *et al.*, 2017; Scheres, 2012). For the $MAT2_4V2_2$ dataset, a total of 10,707 movie stacks were subjected to motion correction using RELION's CPU-based implementation of the motion correction algorithm and initial contrast transfer function (CTF) estimation was performed using CTFFIND-4.1 (Rohou & Grigorieff, 2015). Particle picking was conducted using the pre-trained general model in crYOLO on the high-resolution Krios dataset, and the resulting coordinates were imported into RELION for particle extraction and subsequent processing (**Figure S11**) (Wagner *et al.*, 2019; Scheres, 2012). Approximately 4.5 million particles were initially extracted at a pixel size of 0.74 Å, down sampled and imported into CryoSPARC for 2D classification. A subset of high-quality $MAT2_4V2_2$ (a MAT2 tetramer flanked by a MATV2 dimer; 4:2 model) 2D class averages were selected for *ab-initio* model generation and a few bad classes for decoy model construction, which facilitated further particle cleaning through heterogeneous refinement. During this 2D curation step distinct classes for $MAT2_2V2_1$ (a MAT2 dimer bound to a MATV2 monomer; 2:1 model) were also observed **(Figure S9).**

After heterogenous refinement a 3D volume representing approximately 804,000 particles for $MAT2_4V2_2$ were reextracted to 0.74 Å pixel size and refined to obtain a resolution of 2.5 Å. However, this volume exhibited significant conformational heterogeneity which could not be resolved efficiently by doing 3D classification and local refinement using a mask for either MAT2 or MATV2 in cryoSPARC (Punjani *et al.*, 2017). To address this, the particles were imported into RELION for doing 3D classification without alignment into four distinct classes (Scheres, 2012). Interestingly, one class, comprising ~135,000 particles, displayed well-defined EM density for the MAT2 tetrameric core and the flexible MATV2 at both end albeit at lower resolution. These

particles were re extracted at a pixel size of 0.74 Å and subsequently imported into cryoSPARC for further refinement, including global, local, and non-uniform refinement, ultimately achieving a final resolution of 2.6 Å with C1 symmetry **(Figure S11)** (Punjani *et al.*, 2017). Local resolution estimation was performed in CryoSPARC where the final map of $MAT2_4V2_2$ exhibited a resolution of ~ 2.6 Å in the $MAT2_4$ tetrameric core to ~ 7 Å for one MATV2 subunit the periphery near the so called northern end **(Figure S12)** (Punjani *et al.*, 2017). To mitigate conformational flexibility and enhance resolution, localized signal subtraction was performed using focused masks—one encompassing MATV2 near the southern end and another surrounding the $MAT2_4$ core—followed by local refinement. While this approach did not significantly increase the global resolution, it improved the local map density quality, leading to higher confidence in the rigid-body fitted MATV2 model and enabling more robust structural interpretation. Validation of the EM map such as FSC calculation, viewing direction distribution and orientation diagnostics were performed in CryoSPARC to calculate the resolution and to diagnose preferred orientation **(Figure S13)** (Punjani *et al.*, 2017).

The data processing workflow for the $MAT2_4V1_2$ dataset followed a similar with some modifications **(Figure S14)**. For $MAT2_4V1_{2,}$10,375 movie stacks were motion-corrected using RELION's CPU-based implementation of the motion correction algorithm, and initial CTF estimation was performed using CTFFIND-4.1 (Scheres, 2012; Rohou & Grigorieff, 2015). Particle picking was conducted using the pre-trained general model in crYOLO and the resulting coordinates were imported into RELION for particle extraction and subsequent processing (Wagner *et al.*, 2019; Scheres, 2012). Approximately 10.5 million particles were initially extracted at a pixel size of 0.74 Å, down sampled, and imported into cryoSPARC for 2D classification (Punjani *et al.*, 2017). Distinct 2D class averages for $MAT2_2V1_1$ (a MAT2 dimer bound to a MATV1 monomer; 2:1 model) were again observed which were separated from the rest of the good looking $MAT2_4V1_2$ class average at an early stage **(Figure S9).** High-quality 2D class averages for $MAT2_4V1_2$ were selected for *ab-initio* model generation, while a few poorly resolved classes were used to construct decoy models. Most of the particles (~1,397,240) corresponded to an *ab-initio* model displaying features for both $MAT2_4$ and $MATV1_2$. These particles were further cleaned through iterative 2D classification and heterogeneous refinement, resulting in a well-defined 3D reconstruction.

However, the resulting 3D volume after heterogeneous refinement, comprising ~1,083,066 particles, still required additional curation to improve the local resolution of the MATV1 subunits. To address this, 3D classification without alignment was performed on the entire set of particles using both cryoSPARC and RELION **(Workflow 1, Figure S14)** (Punjani *et al.*, 2017; Scheres, 2012). This yielded one well defined class of 122584 particles (Class II) with distinct features corresponding to a well-resolved $MAT2_4$ tetrameric core and at least one clear MATV1 subunit.

These particles from Class II were re-extracted at a pixel size of 0.74 Å and subsequently imported into cryoSPARC for further iterative refinement, including global, local, and non-uniform refinement with C1 symmetry, ultimately achieving a final resolution of 2.6 Å **(Figure S15)** (Punjani *et al.*, 2017). Local resolution estimation was performed in CryoSPARC where the final map of $MAT2_4V1_2$ exhibited a resolution of ~ 2.5 Å in the $MAT2_4$ tetrameric core to ~ 6 Å for one MATV1 subunit near the so called northern end (Punjani *et al.*, 2017). To address flexibility-driven resolution limitations in MATV1, a focused mask encompassing its southern end was used, followed by local refinement to improve map quality **(Figure S16)**. This step significantly improved the overall resolution and quality of the EM map. Validation of the EM map such as FSC calculation, viewing direction distribution and orientation diagnostics were performed in CryoSPARC to calculate the resolution and to diagnose preferred orientation (Punjani *et al.*, 2017). 3D classification of particles from Class IV led to the identification of a subset of ~51,300 particles in which the MATV1 subunits adopt an anti-conformation i.e. oriented in opposite directions with respect to the $MAT2_4$ core **(Workflow 1, Figure S14)**. Particles belonging to this class were re-extracted using a box size of 380 pixels and refined to a final resolution of 3.1 Å. This class exhibited an attenuated EM density for the MATV1 subunit near the southern end, likely due to increased conformational heterogeneity or flexibility (cFAR=0.05) **(Figure S17)**.

Using a different strategy, focused 3D classification **(Workflow 2 Figure S18)** was employed to further resolve conformational heterogeneity of the $MAT2_4V1_2$ complex. Data processing steps up to the heterogeneous refinement stage followed the same workflow described in **Figure S14**. Following heterogeneous refinement, the dominant class (~1 million particles) featuring a well-defined $MAT2_4$ tetramer and $MATV1_2$ density was subjected to focused 3D classification using a soft mask applied to the southern half of the complex (encompassing $MAT2_2$ and MATV1), yielding six classes. Class I, showing a complete MAT2 tetramer and a well-resolved MATV1 subunit, was selected for further refinement. Particles were re-extracted with a 380-pixel box size, followed by CTF and non-uniform refinement, resulting in a 2.7Å map consistent with the Class II reconstruction from Workflow 1 **(Figure S18)**. Additional classification of Classes II−V revealed alternative conformations, including anti-oriented MATV1 subunits (denoted as #) and disordered MATV1 densities at both ends (denoted £). Separately, Class VI revealed ~95,000 particles with MATV1 subunits adopting a syn-conformation (both subunits oriented in the same direction with respect to $MAT2_4$, denoted as *). These particles were re-extracted (380-pixel box size) and refined to a final resolution of 2.85Å (**Figure S19**).

Cryo-EM data processing enabled clear visualization of both the stable heterohexameric $MAT2_4V2_2$ and $MAT2_4V1_2$ complexes, as well as the more dynamic heterotrimeric forms, $MAT2_2V2_1$ and $MAT2_2V1_1$. Distinct 2D class averages confirmed the coexistence of these

assemblies, consistent with earlier biochemical evidence. Although preferred orientation and pronounced flexibility—particularly within MATV—limited high-resolution 3D reconstruction of the heterotrimers, the results conclusively demonstrate that MAT complexes adopt two stoichiometries, providing new structural insight into their dynamic association states.

3D variability analysis for all reconstructed volumes were performed in CryoSPARC to reveal both discrete and continuous conformational heterogeneity within their final particle sets (Punjani *et al.*, 2017). Results were displayed in simple mode using 20 frames and volume was down sampled to 256 pixel box size. Movies were created by volume morphing method in ChimeraX **(See supplementary movies 1-9)** (Meng *et al.*, 2023).

**Supplementary Table 1: Cryo-EM data collection, refinement and validation statistics**

| **Name** | MAT$\alpha 2_4\beta V1_2$ (Wobbly) | MAT$\alpha 2_4\beta V2_2$ (Wobbly) |
|---|---|---|
| **Data Accession** | | |
| PDB | 9QPP | 9QPO |
| EMDB | 53277 | 53276 |
| **Data Collection** | | |
| Microscope | FEI Titan Krios | FEI Titan Krios |
| Voltage (kV) | 300 | 300 |
| Detector | Falcon 4i | Falcon 4i |
| Energy filter slit width (eV) | 10 | 10 |
| Nominal magnification | 165k | 165k |
| Software | EPU version 3.8 | EPU version 3.8 |
| Pixel size (Å/pixel) | 0.74 | 0.74 |
| Defocus range (m) | -0.6 to -2.4 | -0.6 to -2.4 |
| Exposure time (s) | 2.99 | 2.99 |
| Frames | 40 | 40 |
| Exposure rate ($e^-$ $pixel^{-1}$ $s^{-1}$) | 7.25 | 7.25 |
| Electron exposure ($e^-/Å^2$) | 40 | 40 |
| Dose per frame ($e^-/Å^2$) | 1.0 | 1.0 |
| Micrographs collected | 10375 | 10707 |
| **Reconstruction** | | |
| Software | RELION 4.0.2/ | RELION 4.0.2/ |

| | CryoSPARC 4.6.0 | CryoSPARC 4.6.0 |
|---|---|---|
| Particles used in refinement | 122,584 | 135,219 |
| Symmetry | C1 | C1 |
| Map resolution when FSC=0.143, 0.5 (masked) (Å) | 2.61, 2.8 | 2.60, 2.9 |
| Map sharpening B-factor (Å$^2$) | -94.6 | -91.8 |
| **Model Building and Refinement** | | |
| Software | REFMAC5, Chimera, ChimeraX, Coot | REFMAC5, Chimera, ChimeraX, Coot |
| Non-hydrogen atoms | 14658 | 11801 |
| Protein residues | 1854 | 1515 |
| Water | 173 | 8 |
| Average B factors (Å$^2$) | | |
| Protein | 68 | 125 |
| Ligands and water | 39 | 103 |
| R.M.S. deviations | | |
| Bond length (Å) | 0.005 | 0.004 |
| Bond angle (°) | 0.994 | 0.961 |
| Ramachandran statistics (%) | | |
| Outliers | 0.28 | 0.0 |
| Allowed | 1.98 | 1.74 |
| Favoured | 97.74 | 98.26 |
| MolProbity score | 1.56 | 1.25 |
| ClashScore | 6.2 | 4.76 |
| Poor rotamers (%) | 1.6 | 0.64 |
| Model vs. Map CC (mask) | 0.87 | 0.86 |

**Supplementary Table 2: CryoEM data collection, refinement and validation statistics**

| **Name** | MAT$\alpha 2_4 \beta$V1$_2$ (Syn) | MAT$\alpha 2_4 \beta$V1$_2$ (Anti) |
|---|---|---|
| **Data Accession** | | |
| PDB | 30GD | 30GH |
| EMDB | 57742 | 57756 |
| **Data Collection** | | |

| | | |
|---|---|---|
| Microscope | FEI Titan Krios | FEI Titan Krios |
| Voltage (kV) | 300 | 300 |
| Detector | Falcon 4i | Falcon 4i |
| Energy filter slit width (eV) | 10 | 10 |
| Nominal magnification | 165k | 165k |
| Software | EPU version 3.8 | EPU version 3.8 |
| Pixel size (Å/pixel) | 0.74 | 0.74 |
| Defocus range (m) | -0.6 to -2.4 | -0.6 to -2.4 |
| Exposure time (s) | 2.99 | 2.99 |
| Frames | 40 | 40 |
| Exposure rate ($e^{-}$ $pixel^{-1}$ $s^{-1}$) | 7.25 | 7.25 |
| Electron exposure ($e^{-}$/$Å^{2}$) | 40 | 40 |
| Dose per frame ($e^{-}$/$Å^{2}$) | 1.0 | 1.0 |
| Micrographs collected | 10375 | 10707 |
| **Reconstruction** | | |
| Software | RELION 4.0.2/ CryoSPARC 4.6.0 | RELION 4.0.2/ CryoSPARC 4.6.0 |
| Particles used in refinement | 95,318 | 51,299 |
| Symmetry | C1 | C1 |
| Map resolution when FSC=0.143, 0.5 (masked) (Å) | 2.8, 3.2 | 3.1, 3.6 |
| Map sharpening B-factor ($Å^{2}$) | 97.6 | 88 |
| **Model Building and Refinement** | | |
| Software | REFMAC5, Chimera, ChimeraX, Coot | REFMAC5, Chimera, ChimeraX, Coot |
| Non-hydrogen atoms | 16924 | 16783 |
| Protein residues | 2161 | 2145 |
| Water | - | - |
| Average B factors ($Å^{2}$) | | |
| Protein | 186.46 | 213.2 |
| Ligands and water | - | - |
| R.M.S. deviations | | |
| Bond length (Å) | 0.006 | 0.005 |
| Bond angle (°) | 1.037 | 1.024 |
| Ramachandran statistics (%) | | |
| Outliers | 0.14 | 0.05 |

| | | |
|---|---|---|
| Allowed | 3.13 | 3.39 |
| Favoured | 96.73 | 96.56 |
| MolProbity score | 2.04 | 2.1 |
| ClashScore | 13.2 | 13.94 |
| Poor rotamers (%) | 1.88 | 2.0 |
| Model vs. Map CC (mask) | 0.84 | 0.77 |

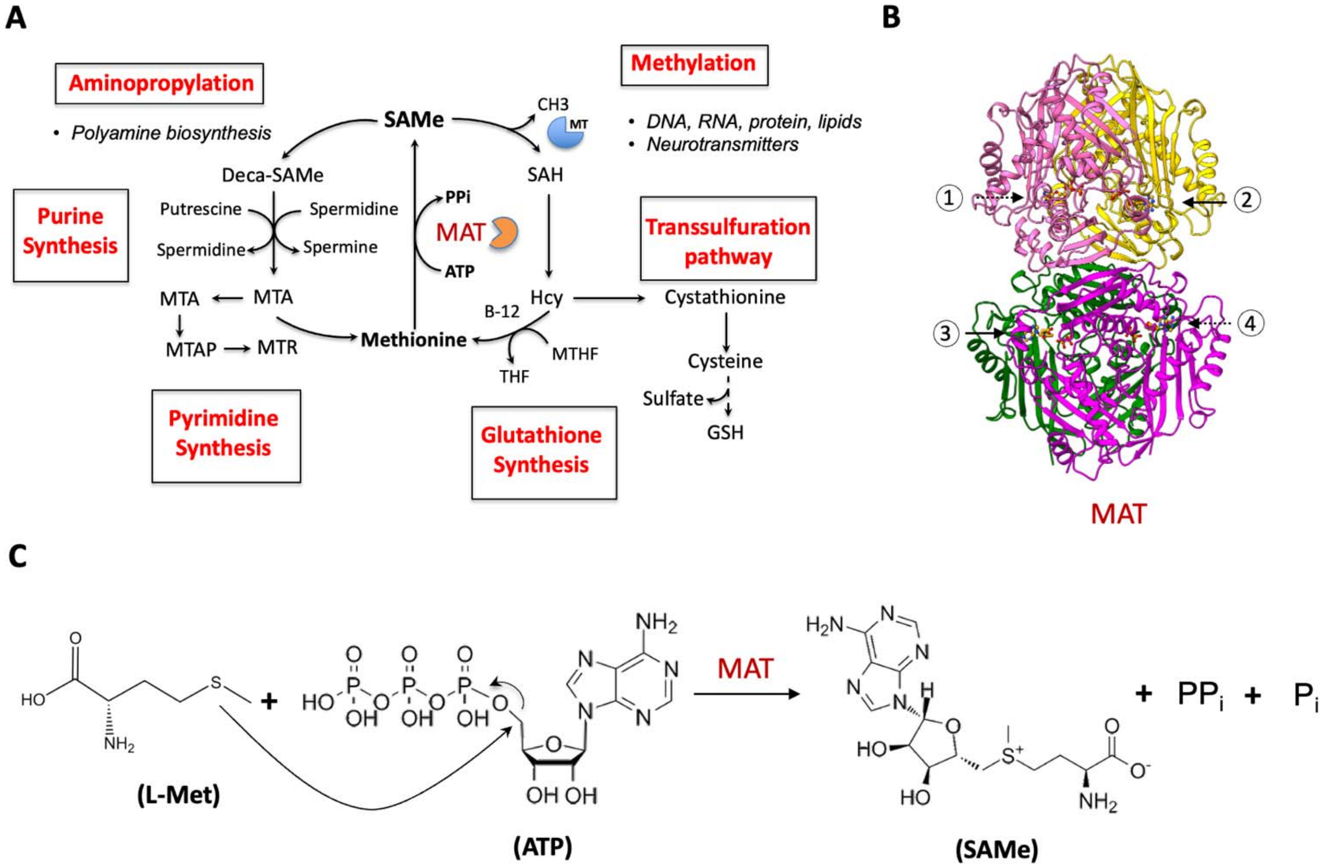


**Figure S1. MAT complexes in SAMe regulation.** **(A)** Overview of the methionine cycle. **(B)** Schematic representation of human methionine adenosyltransferases II complex, highlighting its four active sites. (**C**) Schematic of the enzymatic reaction underlying SAMe synthesis.

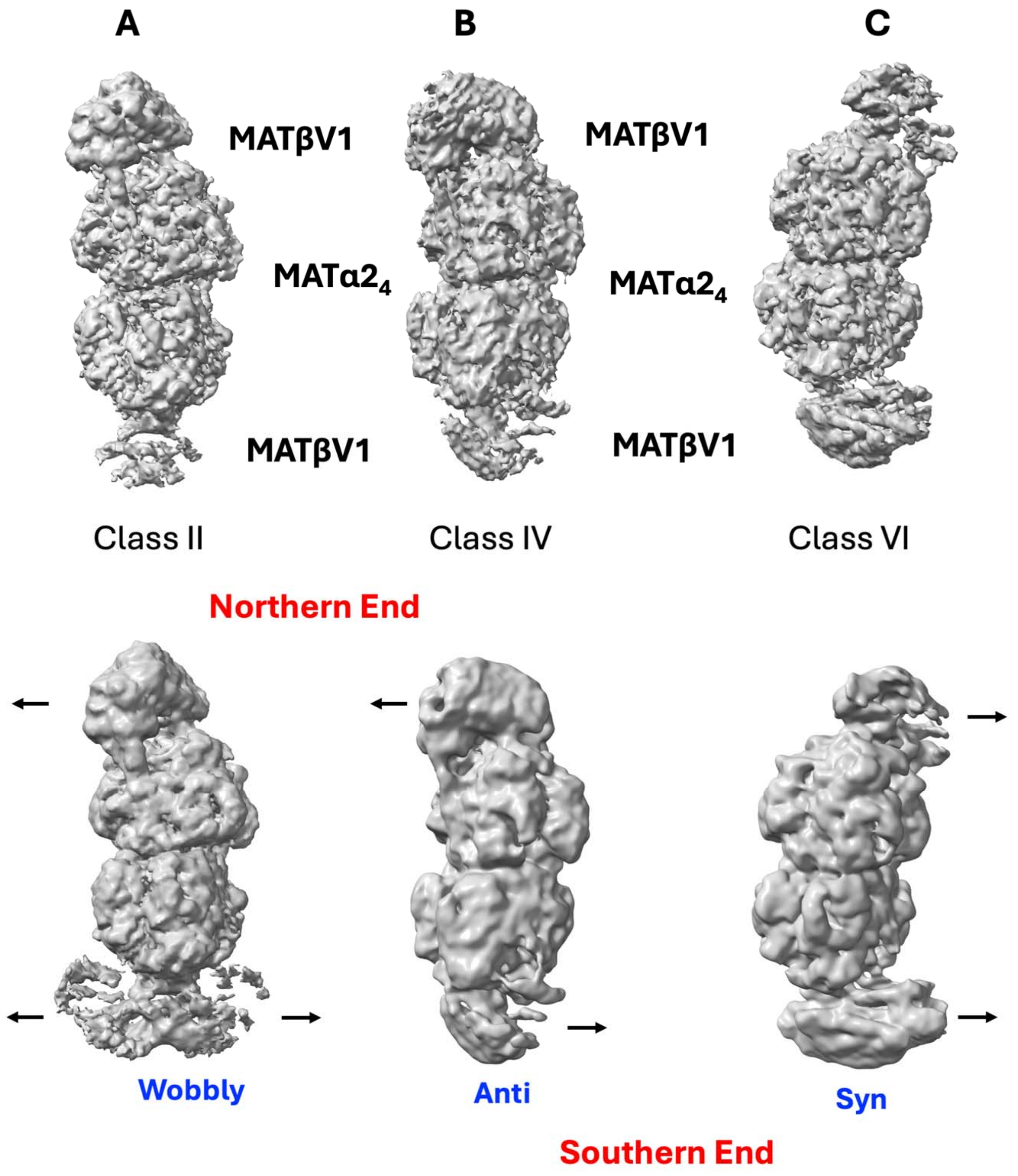


**Figure S2. CryoEM 3D reconstruction of the $MAT\alpha 2_4\beta V1_2$ complex in multiple conformational states. (A)** Class II and **(B)** Class IV were derived from Workflow 1 **(See Figure S14 for more details)**. In Class II, the MATV1 subunits near the southern end adopt a dual or wobbly conformation relative to the $MAT2_4$ core. In Class IV, the MATV1 subunits adopt an anti-conformation near the southern end, with the two subunits oriented in opposite directions relative to the core. **(C)** Class VI, obtained via an alternative focused classification strategy (Workflow 2), exhibits a syn conformation in which both MATV1 subunits near the southern end are aligned in the same direction with respect to the $MAT2_4$ core **(See Figure S18 more details)**. Lower panels show Gaussian-filtered maps (B-factor = 200) corresponding to the map as obtained (unfiltered reconstructions) shown above.

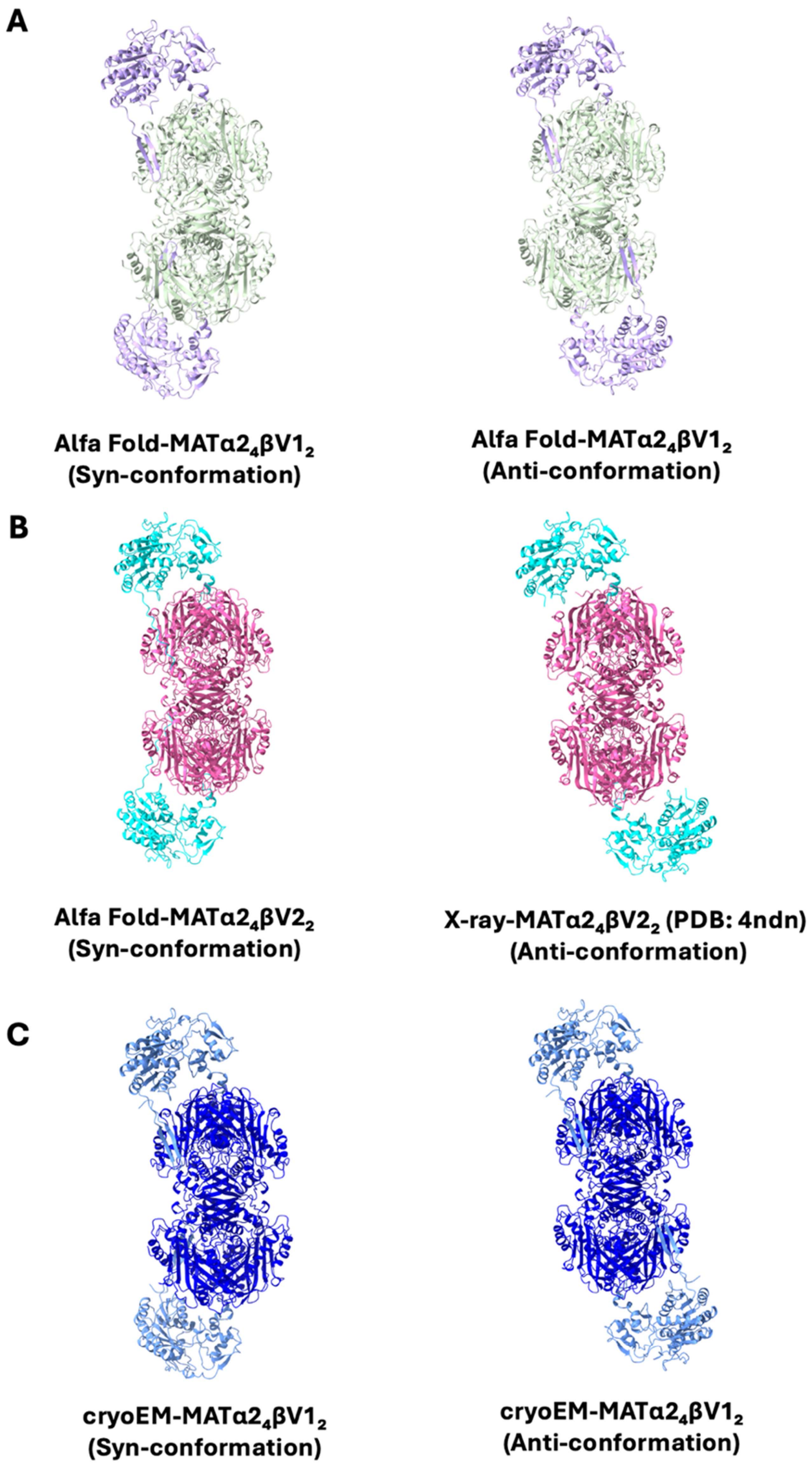


**Figure S3. Conformational diversity of MATα$2_4$βV$_2$ complexes predicted by AlphaFold3 and observed in experimental structures**. **(A)** AlphaFold3 predicts that the MAT$2_4$V$1_2$ complex can adopt both syn and anti-conformations. **(B)** While AlphaFold3 predicts MAT$2_4$V$2_2$ in the syn-conformation, the X-ray crystal structure reveals an anti-conformation. **(C)** CryoEM structures of MAT$2_4$V$1_2$ show the presence of both syn and anti-conformations. These results demonstrate that MAT$2_4$V$1_2$ and MAT$2_4$V$2_2$ complexes exhibit multiple conformational states, with AlphaFold3 predictions closely mirroring the experimentally observed structural heterogeneity.

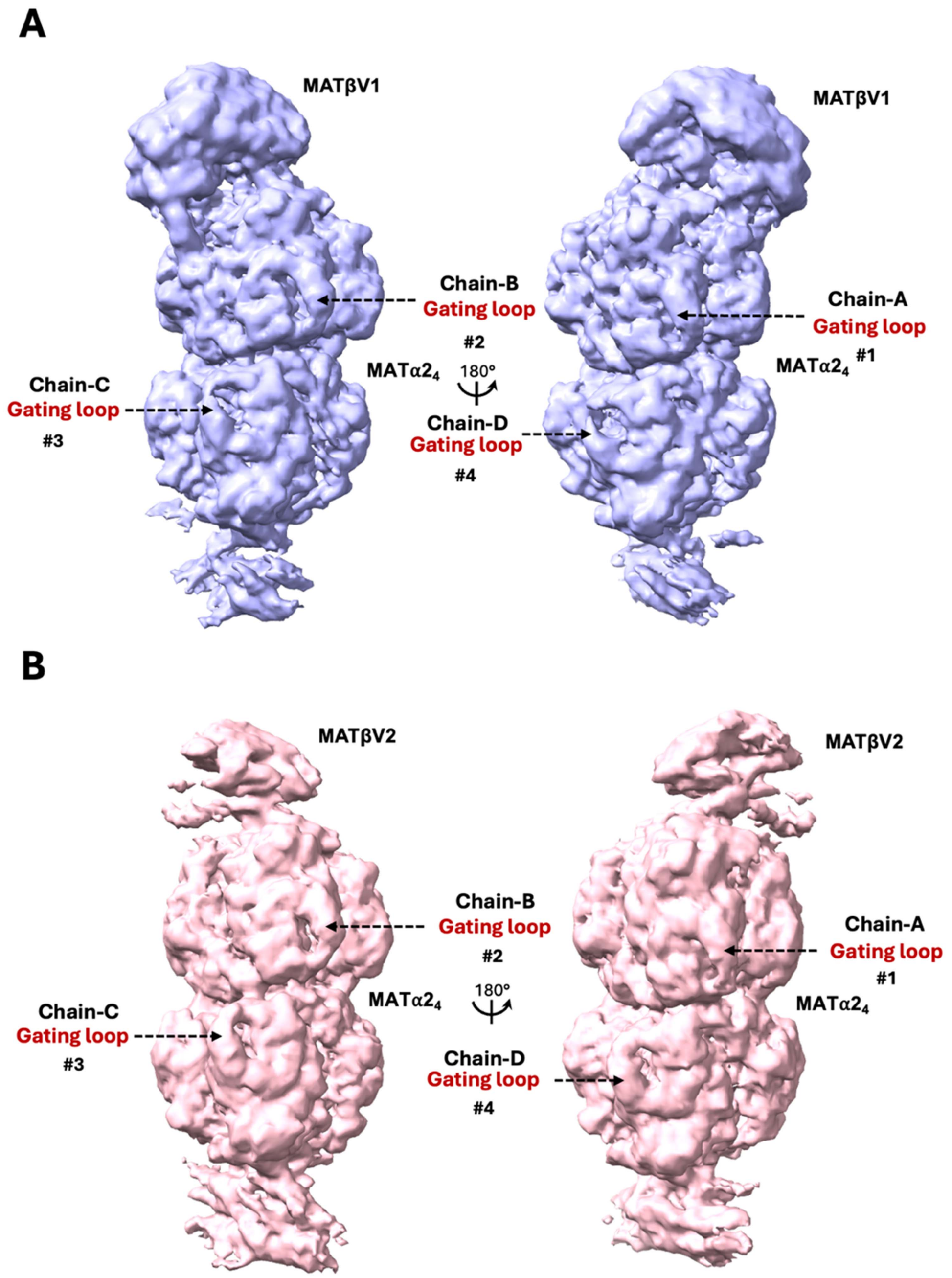


**Figure S4. Gaussian-filtered cryoEM maps of (A)** $MAT2_4V1_2$ and **(B)** $MAT2_4V2_2$ complexes, highlighting the gating loops in the open conformation.

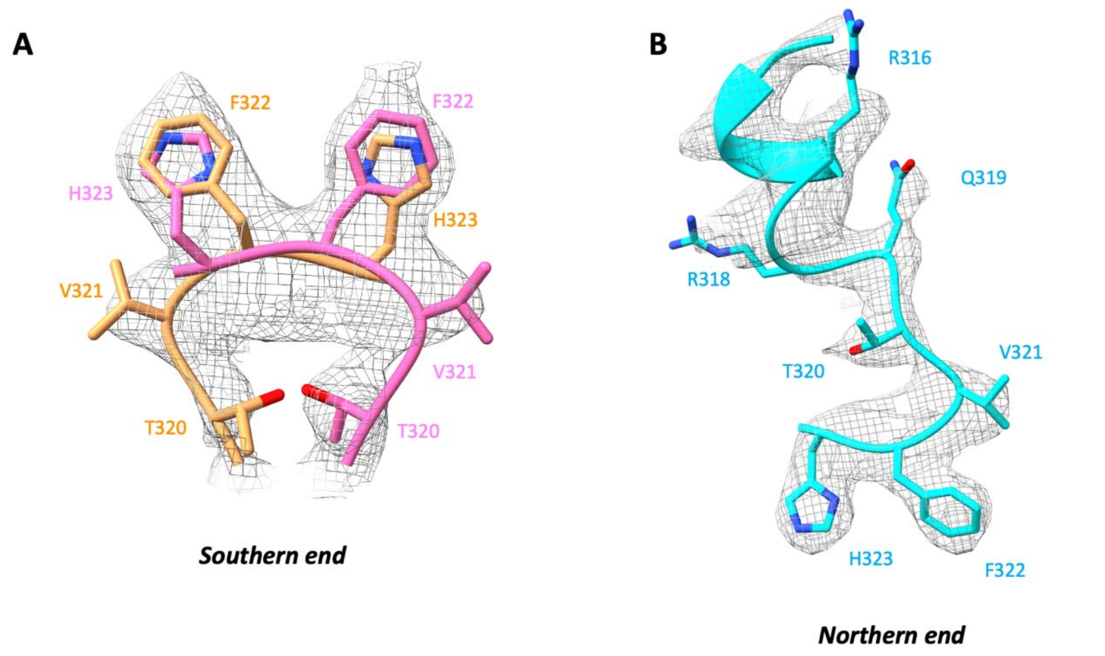


**Figure S5. MATα2 interaction with MATβV2.** The C-terminal residues of MATV2 (Thr320, Val321, Phe322, and His323) insert into the cavity at the MAT2 dimer interface. **(A)** CryoEM density map highlighting two equally probable conformations (50% each) of the four C-terminal residues at the southern end, shown in light orange and magenta. **(B)** The MATV2 EM density at the northern end enabled model building only for the C-terminal residues of (amino acids 311–323).

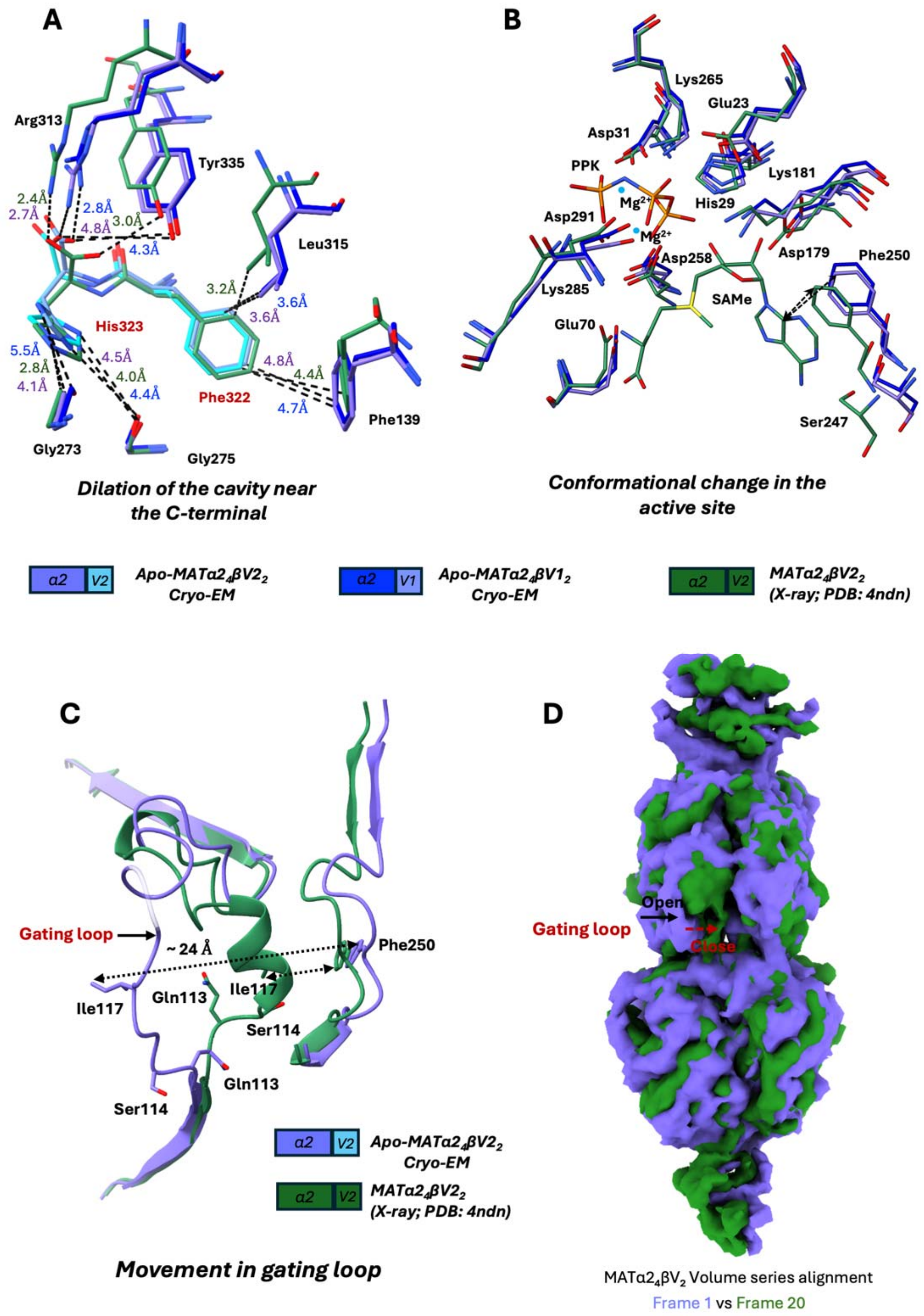


**Figure S6. Structural Comparison of Apo and Product-Bound MATα-MATβV Complexes highlighting structural differences (A)** Close-up of the MATV2-binding interface in the product-bound X-ray structure, showing the widened MAT2 cleft. Distances are indicated by dotted lines and color-coded by structure. MATV residue numbers are labelled in red. **(B)** Active site view of the MAT2 dimer showing key protein residues, SAMe, and the triphosphate analogue PPK as sticks. **(C)** Structural comparison of the gating loop conformation between the cryoEM apo $MAT2_4V2_2$ complex and the SAMe/PPNP-bound $MAT2_4V2_2$ X-ray structure. Structures are color coded as indicated. For cryo-EM apo-$MAT2_4V2_2$, MAT2 is medium slate blue and MATV2 cyan; for SAMe/PNP-bound crystal structure (PDB: 4ndn), MAT2 and MATV2 are green; for cryo-EM apo-$MAT2_4V1_2$, MAT2 is marine blue and MATV1 is shown in cornflower blue. **Structural transition (D)** Depicting the transition between open and closed gating loop conformations in the $MAT2_4V2_2$ complex volume series **(see supplementary movie 9)**.

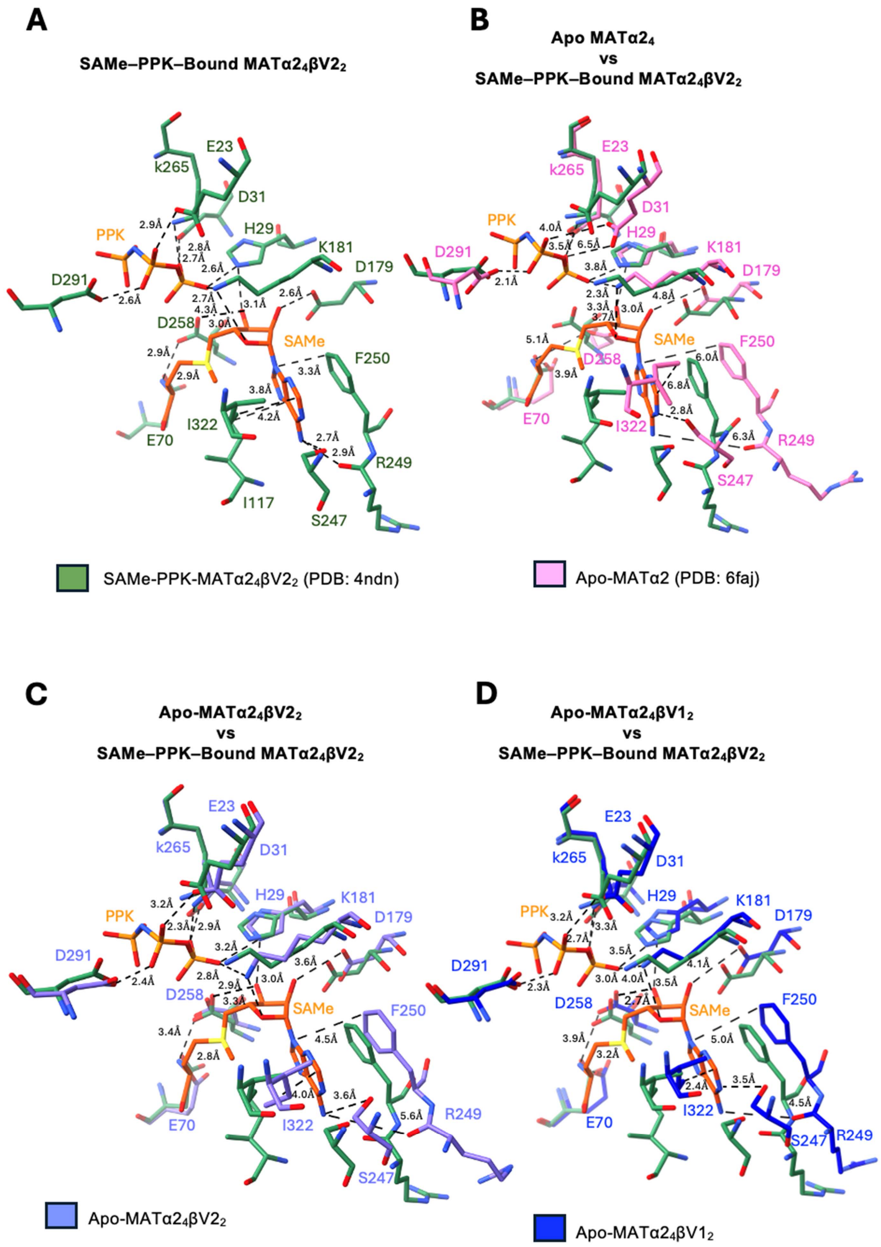


**Figure S7. Structural comparison of MATα2 and MATβV in distinct catalytic states reveals allosteric conformational dynamics.** Shown are the structural transitions of MAT2 in apo and product-bound states, and the regulatory role of MATV isoforms. The green structure represents $MAT2_4V2_2$ bound to SAMe and PPNP (PDB: 4ndn), highlighting the product-bound conformation with MATV2 inserted into the C-terminal cavity of MAT2 **(A)**, while the apo structure of MAT2 (PDB: 6faj) is shown in magenta. Structures from the current study—$MAT2_4V2_2$ and $MAT2_4V1_2$—are depicted in slate and blue, respectively. Chain-specific structural superposition of the MAT2 protomer in the SAMe + PPNP-bound $MAT2_4V2_2$ complex (PDB: 4ndn, Chain B), apo-MAT2 (PDB: 6faj, Chain B), and the resting-state cryoEM structures of $MAT2_4V2_2$ (Chain B) and $MAT2_4V1_2$ (Chain B) reveals conformational shifts in critical active site residues—including His29, Asp179, Lys181, Ser247, Arg249, and Phe250—at equivalent position, demonstrating how MATV binding modulates MAT2's active site architecture. This comprehensive comparison of **(B)** Apo-$MAT2_4$ vs SAMe–PPNP–Bound $MAT2_4V2_2$ **(C)** Apo-$MAT2_4V2_2$ vs SAMe–PPNP–Bound $MAT2_4V2_2$ and **(D)** Apo-$MAT2_4V1_2$ vs SAMe–PPNP–Bound $MAT2_4V2_2$ underscores the dynamic, allosteric regulation exerted by MATV, enhancing the enzymatic efficiency of MAT2 through structural stabilization during the catalytic cycle.

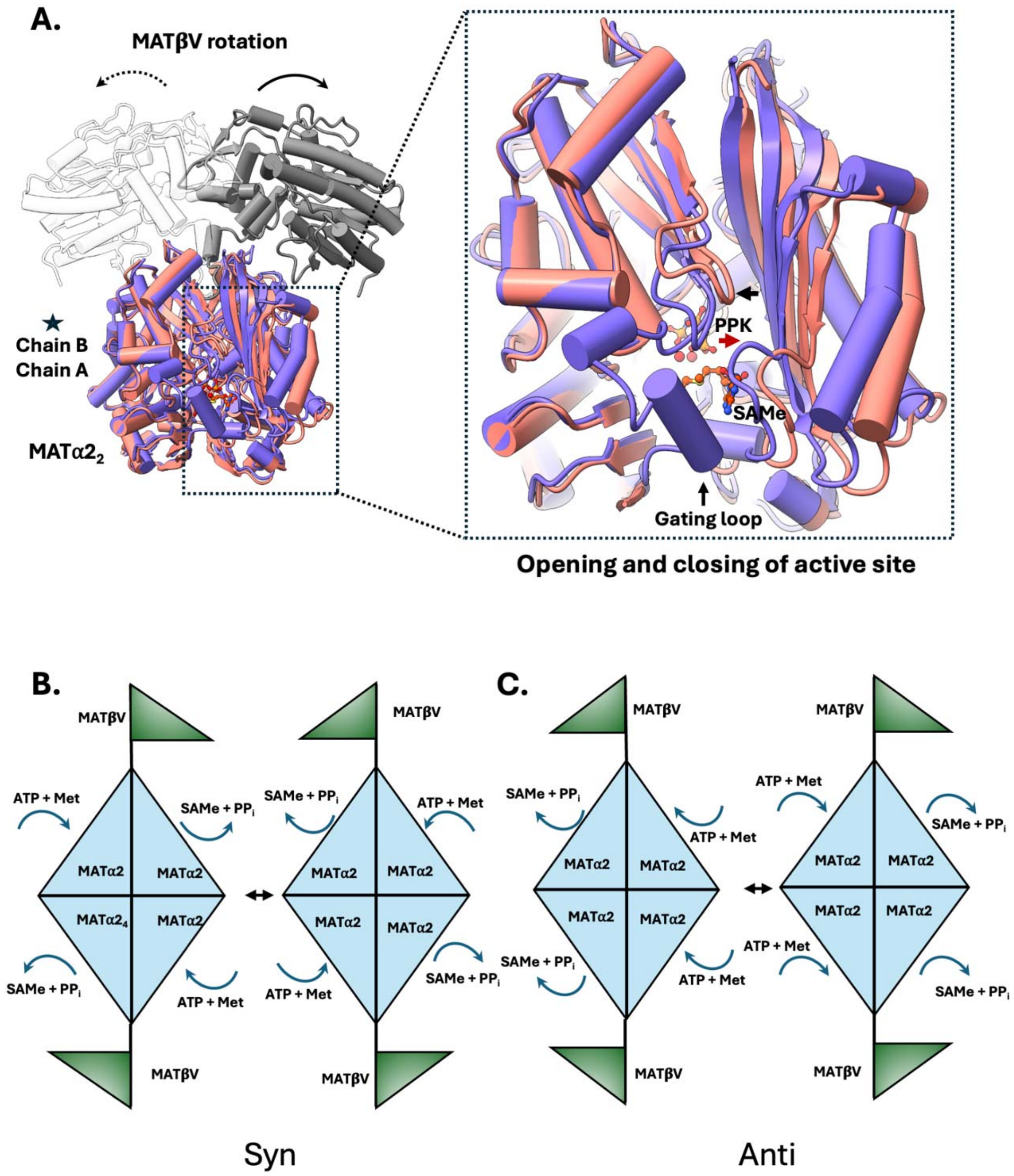


**Figure S8. MATβV rotation modulates the MATα$2_4$ active site for substrate binding and product release. (A)** Chain-wise structural superposition of MAT$2_4$V$2_2$ structures (PDB: 4ndn; Chain A and Chain B), was performed to examine differences in the gating loop and active site architecture during MATV2 rotation. As MATV2 rotates across the MAT$2_4$ dimeric interface, the gating loop shifts from a closed conformation in the ligand-bound state to an open, disordered loop in the apo state, highlighting the functional importance of MATV rotation during the catalytic cycle. **(B, C)** Based on cryo-EM 3D reconstructions of the MAT–MATV complex captured in multiple conformational states, we propose that MATV functions as an allosteric regulator, whose structural flexibility facilitates catalysis by modulating substrate entry, binding, and product release, with each orientation (syn or anti) potentially favoring a distinct step of the reaction.

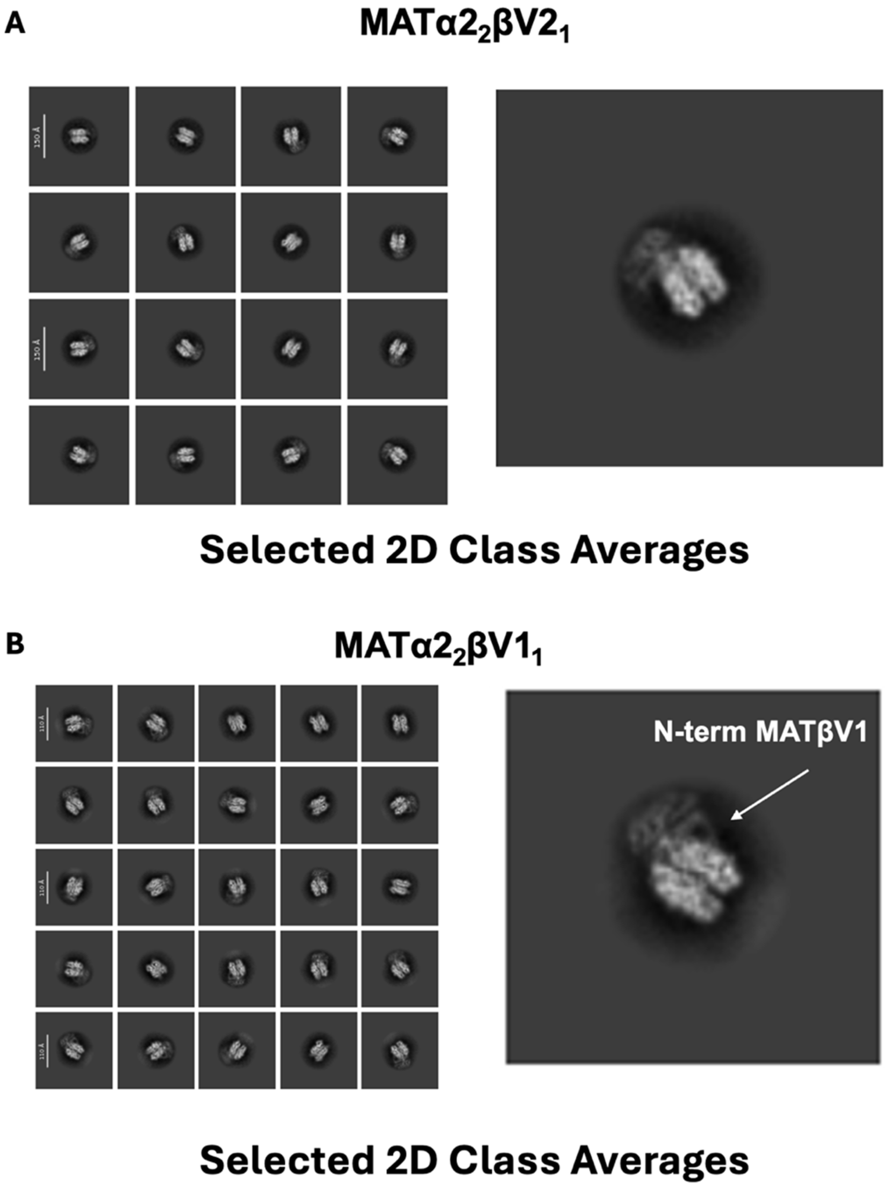


**Figure S9. Selected 2D class averages of the heterodimeric MATα2$_2$βV2$_1$ and MATα2$_2$βV1$_1$ complex. (A, B)** The figures display representative 2D class averages from cryo-EM data processing, highlighting the structural features of the MAT2$_2$V2$_1$ and MAT2$_2$V2$_1$ offering evidence of the heterodimeric complex formations.

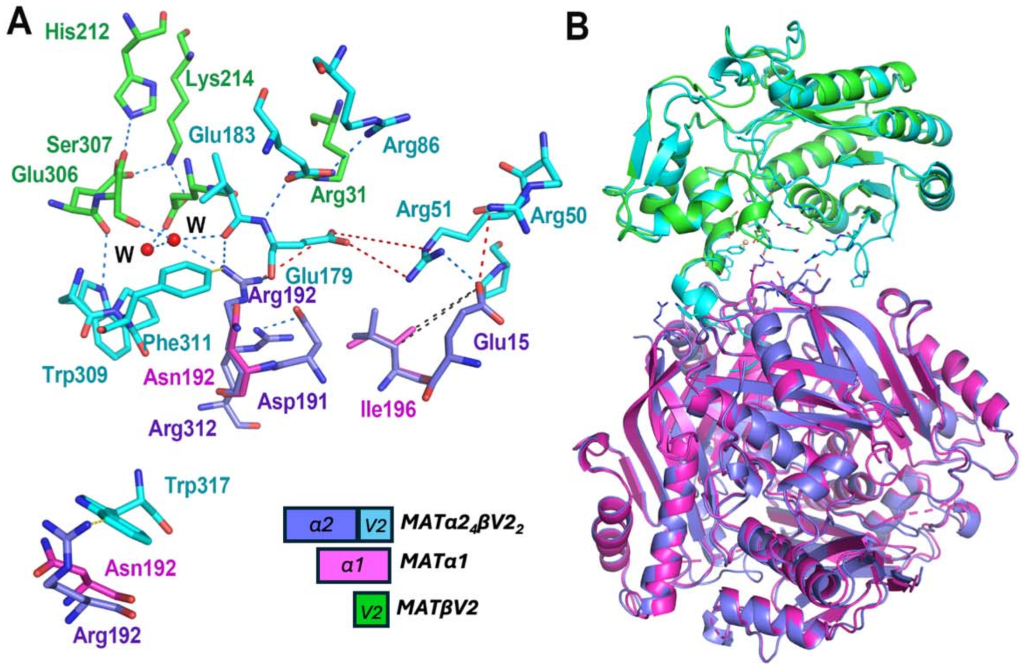


**Figure S10. Structural Insights into MATα1βV2 complex formation reveal selective interaction with MATβV1 over MATβV2.** In MAT2, binding to MATV is mediated through two major interaction modules: the C-terminal region of MATV, which inserts into the MAT dimeric interface, and the N-terminal -hairpin present only in MATV1, which interacts with a hydrophobic surface patch on MAT2. Because MATV1 contains both elements, it binds more strongly than MATV2, which relies solely on its C-terminal tail. Consistently, ITC experiments show that both MAT1 and MAT2 have higher affinity for MATV1 than for MATV2, and the V_max of MAT2 increases by ~34% in the presence of MATV1, indicating that N-terminal differences directly influence complex stability and catalytic activity (Panmanee *et al.*, 2020). Despite >80% sequence identity with MAT2, MAT1 forms a stable complex mainly with MATV1 and interacts only weakly with MATV2 (Murray *et al.*, 2014). The structure of the MAT1−MATV complexes remains unknown, but insights can be drawn from structural analyses of MAT2 complexes. To investigate this discrepancy, in silico models were generated by replacing MAT2 with MAT1 in previously determined MAT2 complexes. **(A)** MAT1 (PDB: 6sw5, magenta) was superimposed onto the MAT2 subunit within the apo MAT2$_4$V1$_2$ complex (PDB: 4ndn) to generate the MAT1$_4$V1$_2$ model. **(B)** Similarly, MAT1 was superimposed onto the MAT2 subunit within the apo and product-bound MAT2$_4$V2$_2$ complex (PDB: 4ndn) to generate the MAT1$_4$V2$_2$ model. Pairwise structural superposition of the MATV2 crystal structure (PDB: 2ydy, green) onto MATV2 within the product-bound MAT2$_4$V2$_2$ complex was performed to compensate for poorly resolved regions in the cryo-EM and ligand-bound X-ray structures. The resulting MAT1 models displayed no steric clashes and preserved the expected binding positions of the MATV1 C-terminal tail and -hairpin. In contrast, MATV2 binding is predicted to be weaker due to the absence of the -hairpin and local MAT1 substitutions (Arg192Asn, Ile196Leu), which disrupt key ionic, cation−π, and hydrophobic interactions required for MAT2−MATV2 stability. These structural features explain why MAT1 robustly associates with MATV1 in vitro but only weakly or transiently with MATV2.

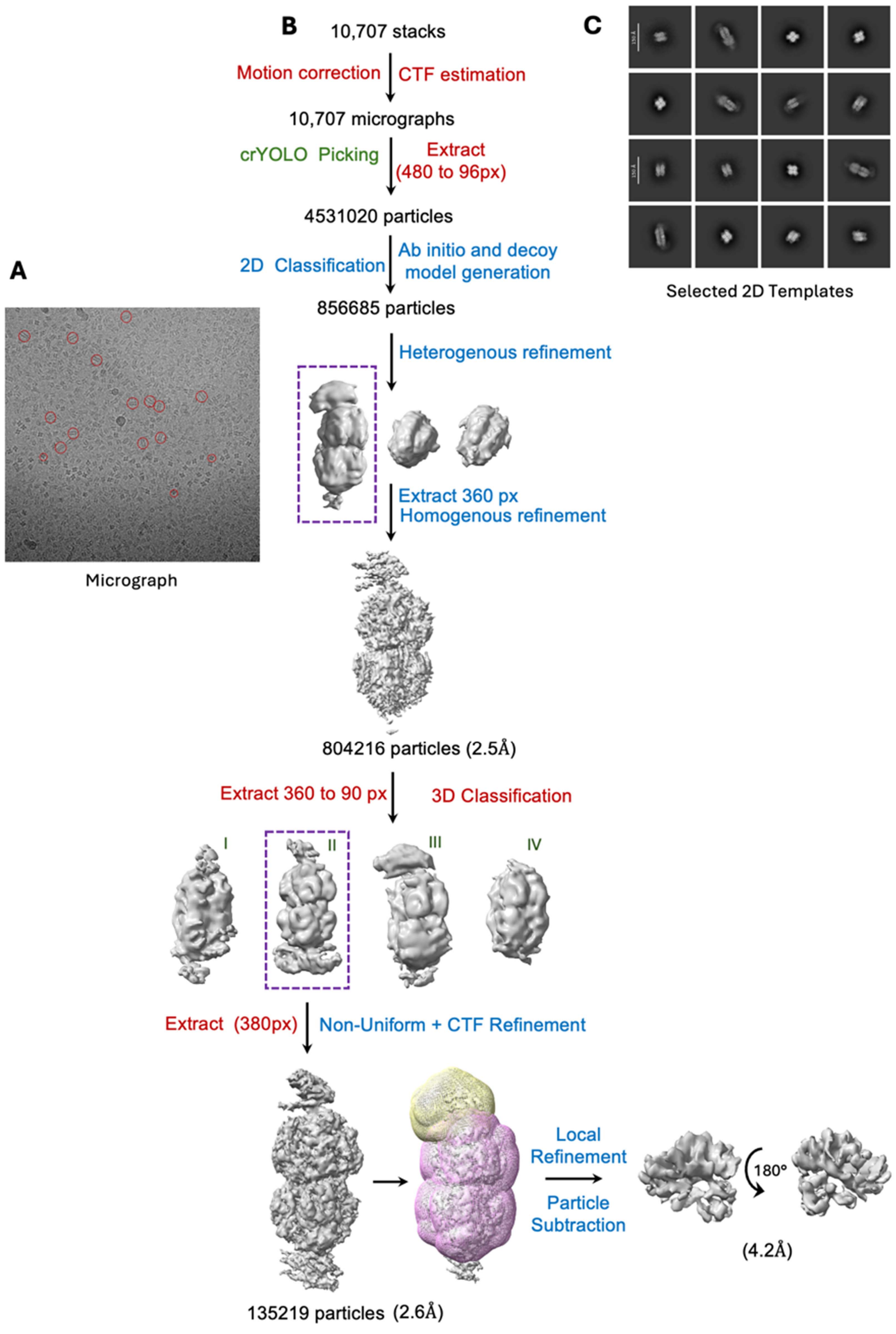


**Figure S11: Processing workflow for the single-particle cryo-EM analysis of Apo-MATα2$_4$βV2$_2$. (A)** Representative micrograph (left) showing MAT2$_4$V2$_2$ particles in red circles. **(B)** Data processing was performed using an integrative approach, combining RELION 4.0 (steps shown in red) and CryoSPARC (steps shown in blue), unless stated otherwise. Motion correction and initial CTF estimation were carried out using RELION's own implementation and CTFFIND 4.1, while crYOLO (step shown in green) was used for particle picking. The particles were extracted with 4 binning, followed by 2D classification to generate templates for ab initio model building and decoy model generation for subsequent 3D curation steps. Following

heterogeneous refinement, a major class was identified that exhibited the $MAT2_4$ tetrameric core along with partial $MATV2_2$ features. This class was selected for further 3D classification (without alignment, four classes). A well-defined class comprising 135219 particles (Class II) displaying both the $MAT2_4$ tetrameric core and $MATV2_2$ at both the northern and southern regions was subsequently re-extracted using a box size of 380 pixels and further refined using CTF refinement and non-uniform refinement, yielding a final resolution of 2.61Å. To enhance structural detail, a focused mask was applied to $MATV_2$ at the northern region (yellow) and $MAT2_4$ (magenta), enabling particle subtraction and localized refinement. This approach improved the resolution and visibility of $MATV_2$ at the northern end to approximately 4.2Å. **(C)** Selected 2D class averages of $MAT2_4V2_2$.

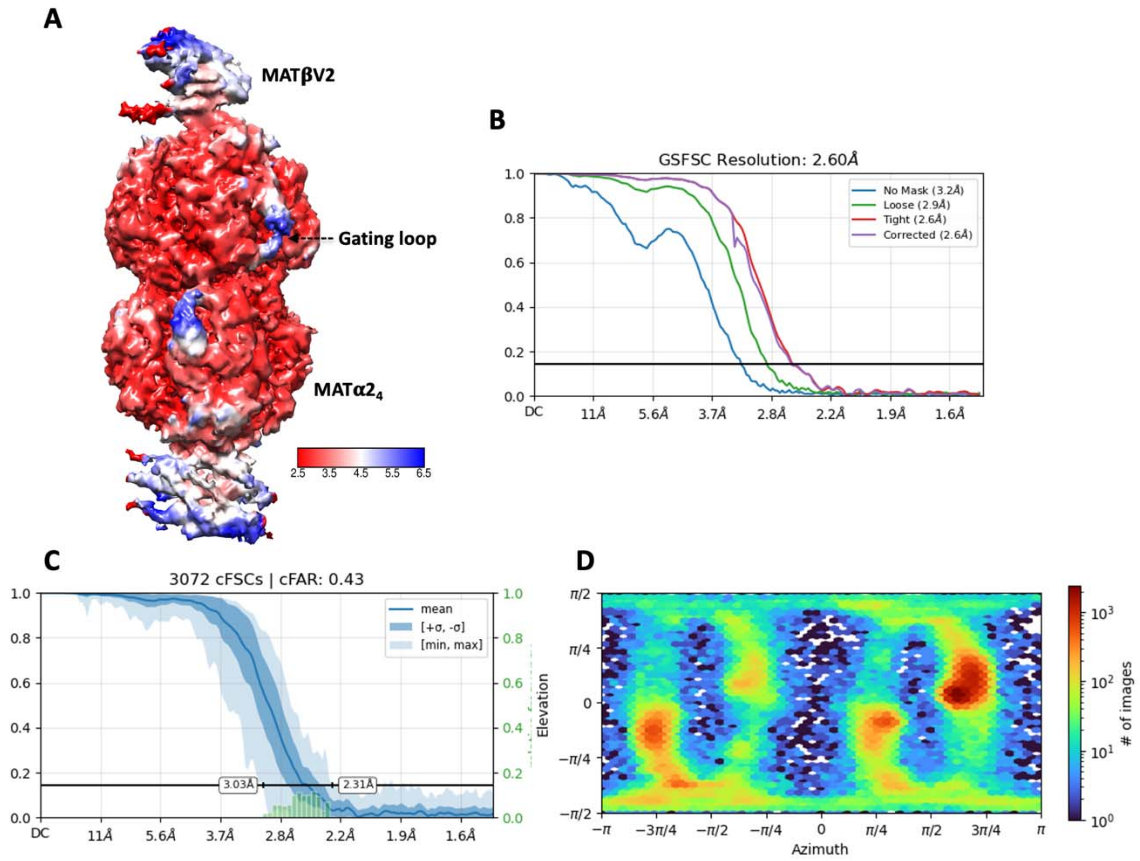


**Figure S12. FSC curve, Local resolution analysis, cFAR plot and viewing distribution plot for $MAT\alpha2_4\beta V2_2$ reconstruction. (A)** Local resolution distribution map, showing a resolution of ~ 2.60Å for the $MAT2_4$ tetrameric core, while the gating loop and one $MATV_2$ subunit near the northern end exhibit a lower resolution of ~ 6-7.0Å. **(B)** cryoPARC generated Gold-standard Fourier shell correlation (GSFSC) curve for the final map, indicating an overall resolution of 2.60Å based on the 0.143 FSC cut-off criterion. **(C)** Conical Fourier Amplitude Ratio (cFAR) plot illustrating the directional distribution of the signal content within particles. cFAR values below 0.5 suggest the presence of preferred orientation. **(D)** Viewing direction distribution histogram, depicting the number of particle images assigned to each viewing direction at different elevations.

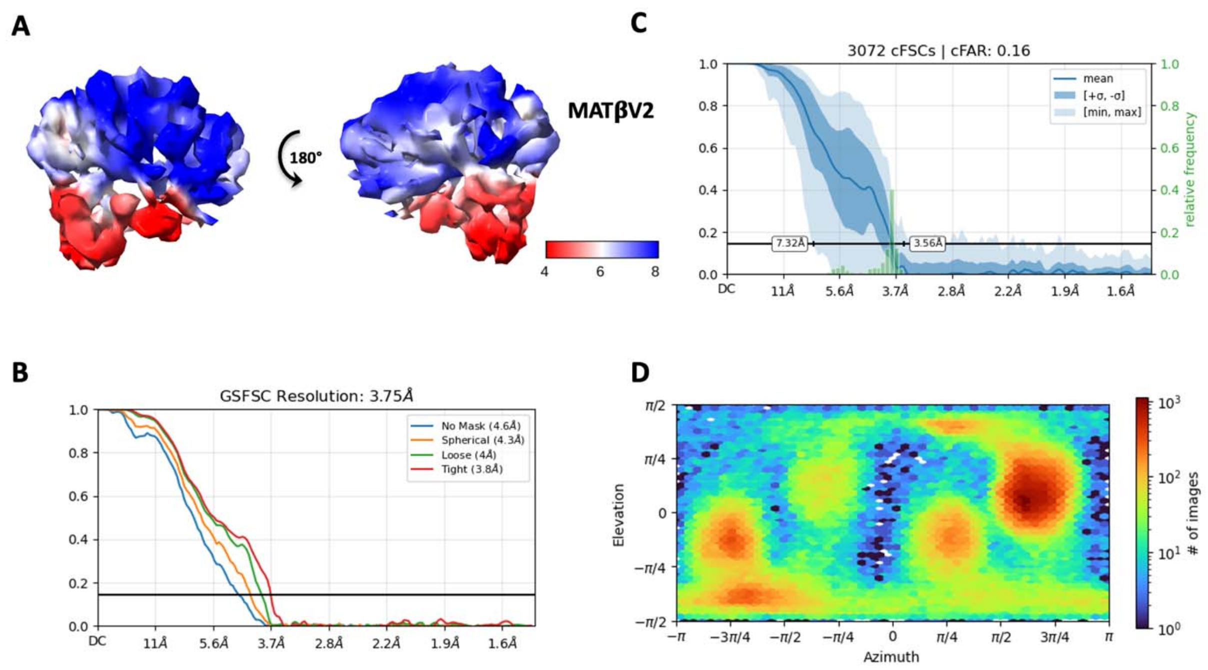


**Figure S13. Local resolution analysis, FSC curve, cFAR plot and viewing distribution plot for MATβV2 after local refinement near the northern end. (A)** Local resolution distribution map, showing a resolution of ~ 4.0Å for the MATV2 near its C-terminal end to ~ 7-8.0Å near periphery. **(B)** Gold-standard Fourier shell correlation (GSFSC) curve for the final map, indicating an overall resolution of 3.75Å based on the 0.143 FSC cut-off criterion. **(C)** Conical Fourier Amplitude Ratio (cFAR) plot illustrating the directional distribution of the signal content within particles. cFAR values below 0.5 suggest the presence of preferred orientation. **(D)** Viewing direction distribution histogram, depicting the number of particle images assigned to each viewing direction at different elevations.

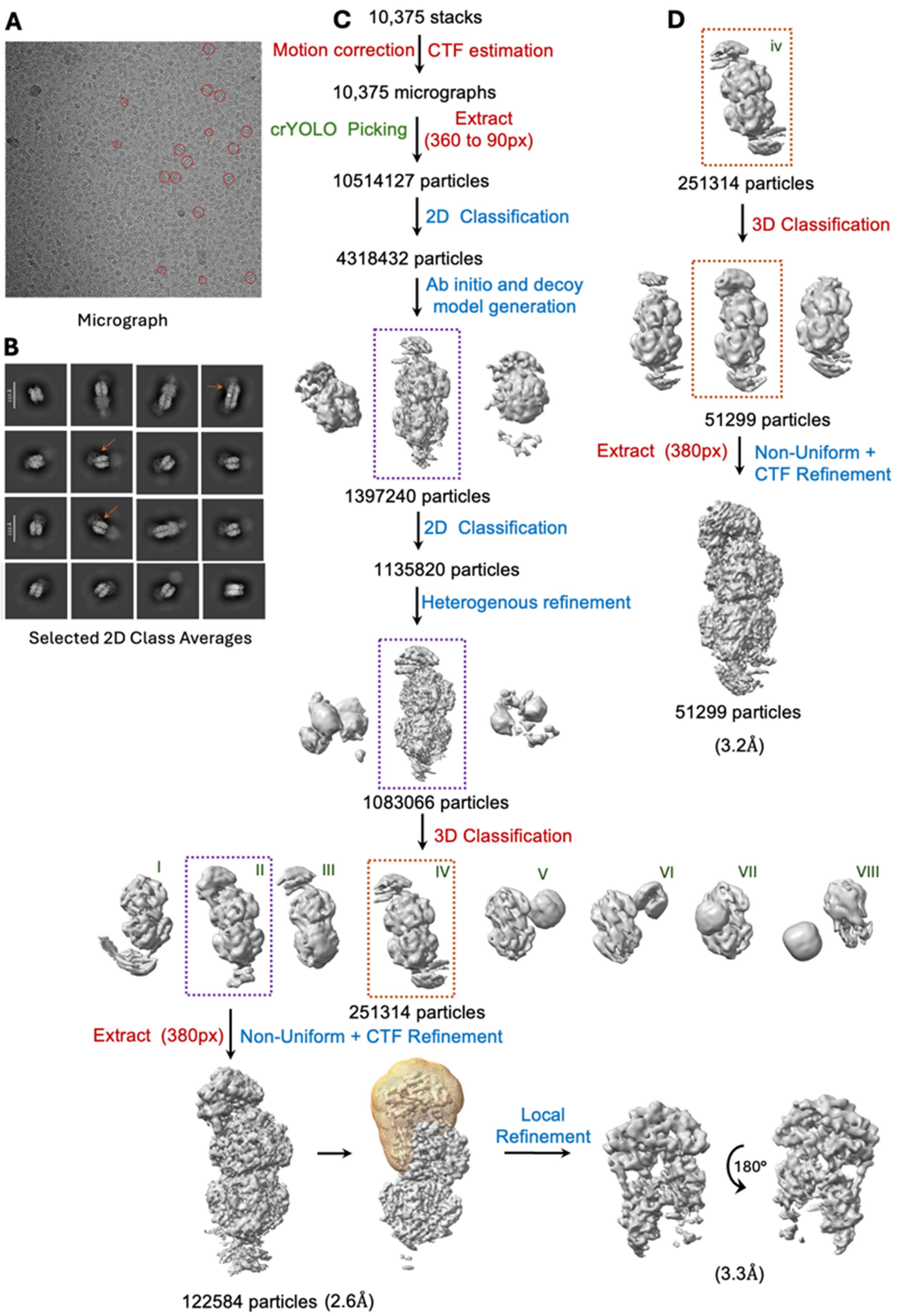


**Figure S14: Processing workflow 1 for the single-particle cryo-EM analysis of Apo-MATα2$_4$βV1$_2$: (A)** Representative micrograph (left) showing MAT2$_4$V1$_2$ particles in red circles. **(B)** Selected 2D class averages of MAT2$_4$V1$_2$, highlighting the N-terminal -hairpin loop of MATV1 (indicated by arrows). **(C)** Data processing was performed using an integrative approach, combining RELION 4.0 (steps shown in red) and CryoSPARC (steps shown in blue), unless stated otherwise. Motion correction and initial CTF estimation were carried out using RELION's own implementation and CTFFIND 4.1, while crYOLO (step shown in green) was used for particle picking. The particles were extracted with 4 binning, followed by 2D classification to generate templates for ab initio model building. A major class from the ab initio modelling step represented MAT2$_4$V1 (~ 1.4 million particles) which was further curated by performing 2D classification and one round of heterogenous refinement to remove bad particles. A major class identified from heterogeneous refinement (~1 million particles) exhibited the MAT2$_4$ tetrameric

core along with MATV1 $_2$ features and was selected for 3D classification (without alignment, eight classes). A well-defined class representing ~122584 particles (Class II) displaying both the MAT2$_4$ tetrameric core and a complete MATV1 at its northern end was re-extracted using a box size of 380 pixels. Subsequent processing, including CTF refinement and non-uniform refinement, resulted in a final resolution of 2.6Å. To further enhance structural detail, a focused mask was applied to MATV1 at its northern end (yellow) for local refinement, improving its visibility and increasing its resolution to approximately 3.3Å. **(D)** 3D classification of particles from Class IV led to the identification of a subset of ~51,300 particles in which the MATV1 subunits adopt an anti-conformation i.e., oriented in opposite directions with respect to the MAT2$_4$ core. Particles belonging to this class were re-extracted using a box size of 380 pixels and refined to a final resolution of 3.2 Å.

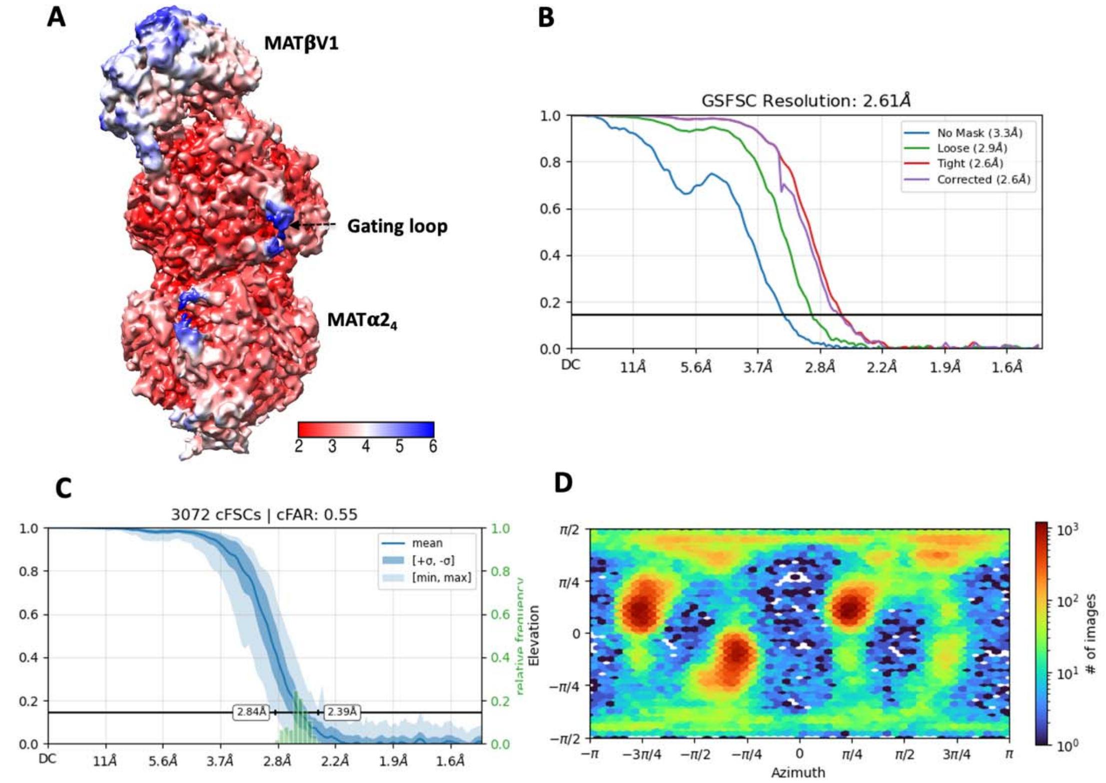


**Figure S15. FSC curve, Local resolution analysis, cFAR plot and viewing distribution plot for MATα2$_4$βV1$_2$ reconstruction. (A)** Local resolution distribution map, showing a resolution of ~ 2.60Å for the MAT2$_4$ tetrameric core, while the gating loop and one MATV1 subunit near the northern end exhibit a lower resolution of ~ 6Å. **(B)** Gold-standard Fourier shell correlation (GSFSC) curve for the final map, indicating an overall resolution of 2.60Å based on the 0.143 FSC cut-off criterion. **(C)** Conical Fourier Amplitude Ratio (cFAR) plot illustrating the directional distribution of the signal content within particles. cFAR values below 0.5 suggest the presence of preferred orientation. **(D)** Viewing direction distribution histogram, depicting the number of particle images assigned to each viewing direction at different elevations.

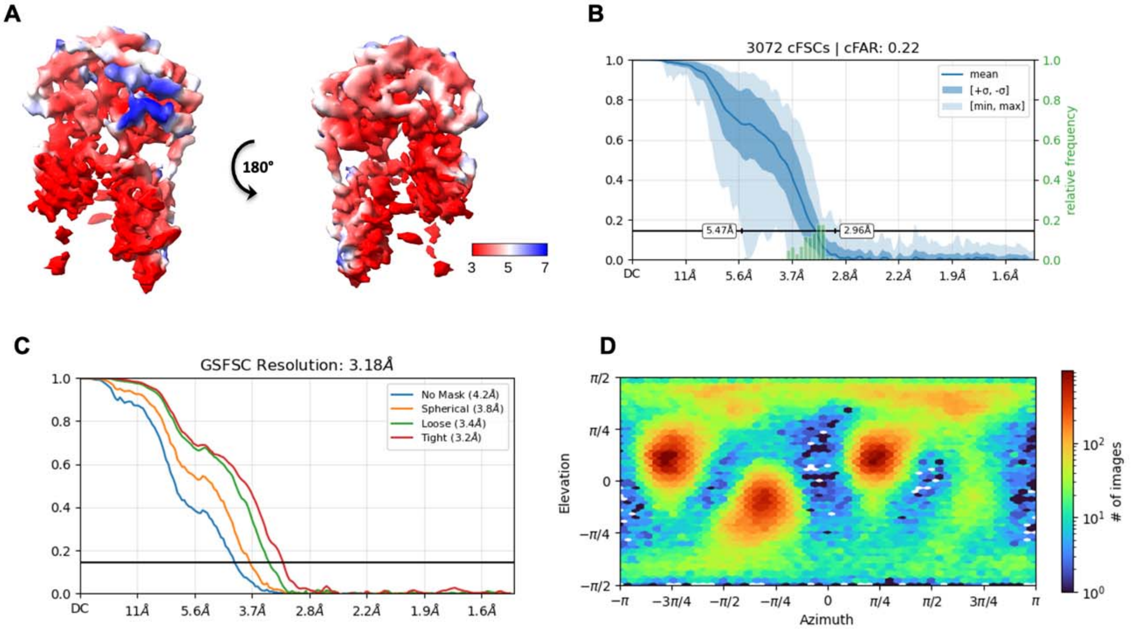


**Figure S16. Local resolution analysis, FSC curve, cFAR plot and viewing distribution plot for MATβV1 after local refinement near the northern end. (A)** Local resolution distribution map, showing a resolution of ~ 3-4.0Å for the MATV1 near its C-terminal and N-terminal end to ~ 5.0Å near periphery. **(B)** Gold-standard Fourier shell correlation (GSFSC) curve for the final map, indicating an overall resolution of 3.2Å based on the 0.143 FSC cut-off criterion. **(C)** Conical Fourier Amplitude Ratio (cFAR) plot illustrating the directional distribution of the signal content within particles. cFAR values below 0.5 suggest the presence of preferred orientation. **(D)** Viewing direction distribution histogram, depicting the number of particle images assigned to each viewing direction at different elevations.

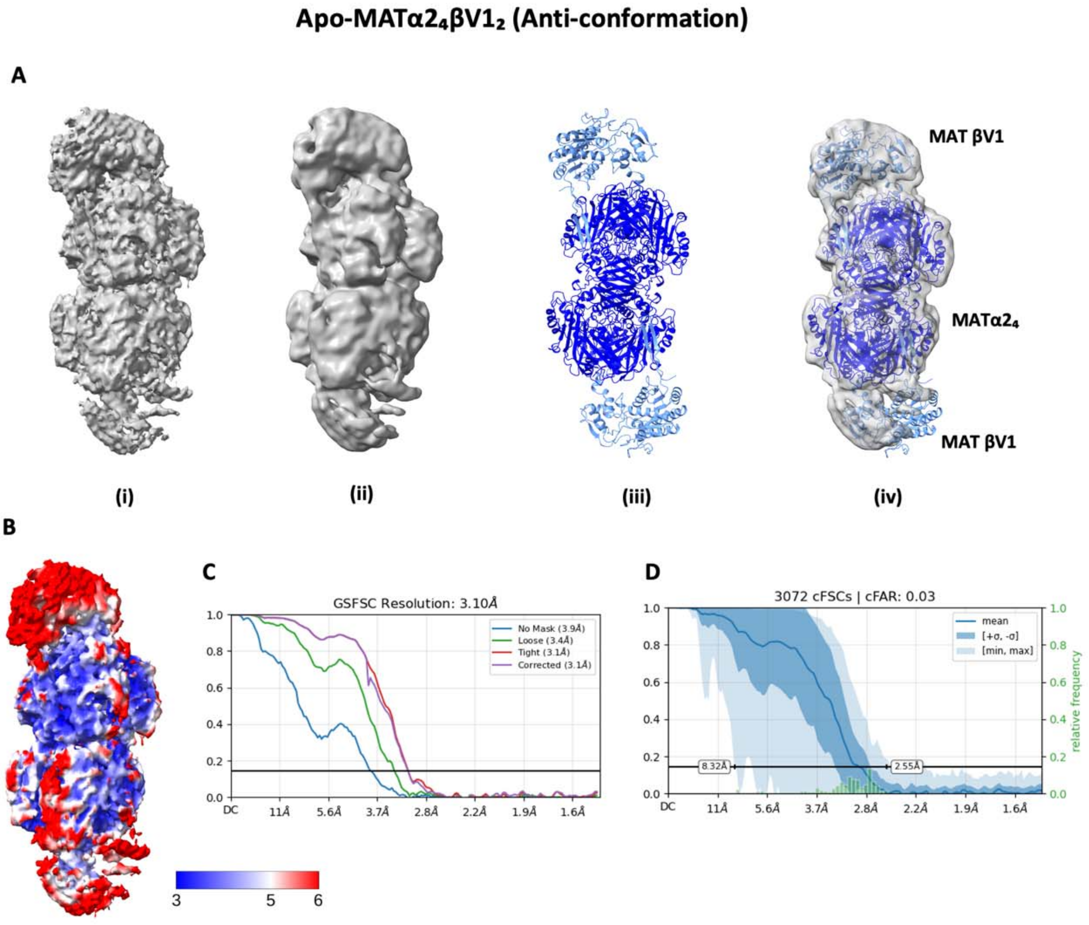


**Figure S17. Model building, FSC curve, cFAR plot, viewing direction distribution, and local resolution analysis for $MAT\alpha 2_4\beta V1_2$ in the anti-conformation state.** A) CryoEM analysis of the $MAT2_4V1_2$ complex in the anti-conformation state: **(i)** as-obtained cryoEM map; **(ii)** Gaussian-filtered map with a B-factor of 200; **(iii)** $MAT2_4V1_2$ model showing $MATV1_2$ in anti-conformation; **(iv)** rigid-body fitting of $MAT2_4V1_2$ complex into the Gaussian-filtered map. **B)** Local resolution map showing ~3.0-4.0Å resolution in the $MAT2_4$ tetrameric core and ~6Å resolution for the MATV1 subunit near the ends. **C)** Gold-standard Fourier Shell Correlation (GSFSC) curve for the final reconstruction, indicating a global resolution of 3.10Å at the 0.143 FSC cut-off. **D)** Conical Fourier Amplitude Ratio (cFAR) plot showing the directional distribution of signal within particles.

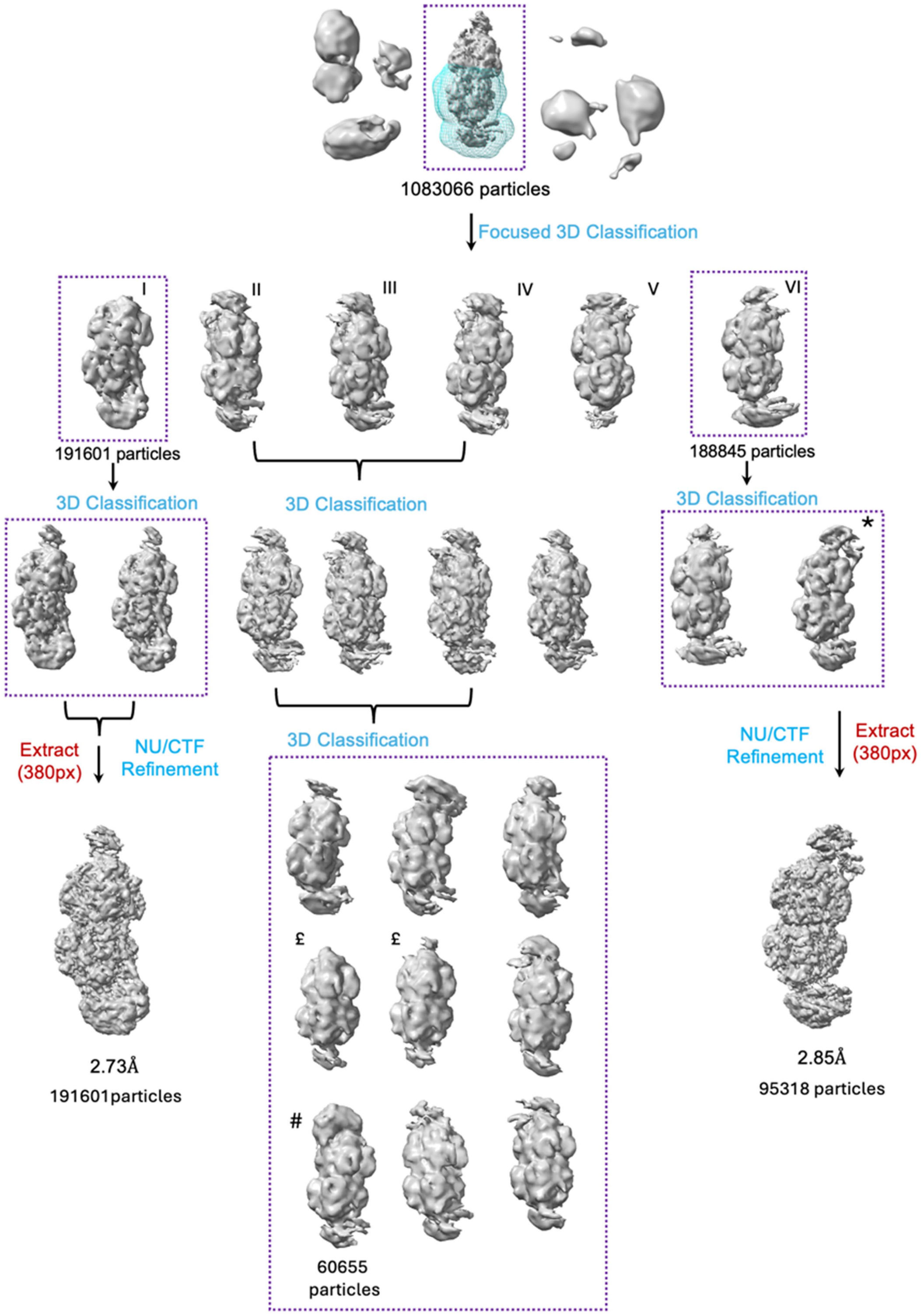


**Figure S18: Processing workflow 2 to resolve conformational heterogeneity of MATα2$_4$βV1$_2$ complex via focused 3D Classification and high-resolution CryoEM refinement:** The data processing steps up to the heterogeneous refinement stage followed the same workflow described in **Figure S14**. The major class (~1 million particles) emerging from heterogeneous refinement, featuring the MAT2$_4$ tetrameric core along with well-defined MATV1$_2$ density, was selected for focused 3D classification. A soft mask was applied to the southern half of the MAT2$_4$V1$_2$ complex, specifically targeting the MAT2$_2$ and MATV1 region, and six classes were generated. Among these, a well-defined class (Class I) displaying both the complete MAT2$_4$

tetramer and a well-resolved MATV1 subunit at the southern end was identified. Particles from this class were re-extracted using a 380-pixel box size and further processed through CTF refinement and non-uniform refinement, resulting in a final map at 2.7Å resolution. The features and map quality closely matched those of the Class II reconstruction of the $MAT2_4V1_2$ complex previously reported in Supplementary Figure 12. Subsequent 3D classifications were performed by combining particles from Classes II, III, IV, and V. These analyses revealed specific classes of $MAT2_4V1_2$ particles in which the MATV1 subunits adopt an anti-conformation (indicated with a superscript hash symbol, #) and some classes where MATV1 at both ends were disordered (indicated with superscript star symbol, £). In parallel, 3D classification of particles from Class VI identified a distinct subset of ~95,000 particles in which MATV1 subunits adopt a syn conformation, i.e., oriented in the same direction relative to the $MAT2_4$ core (indicated by asterisks). These particles were also re-extracted using a 380-pixel box size and refined to a final resolution of 2.85Å.

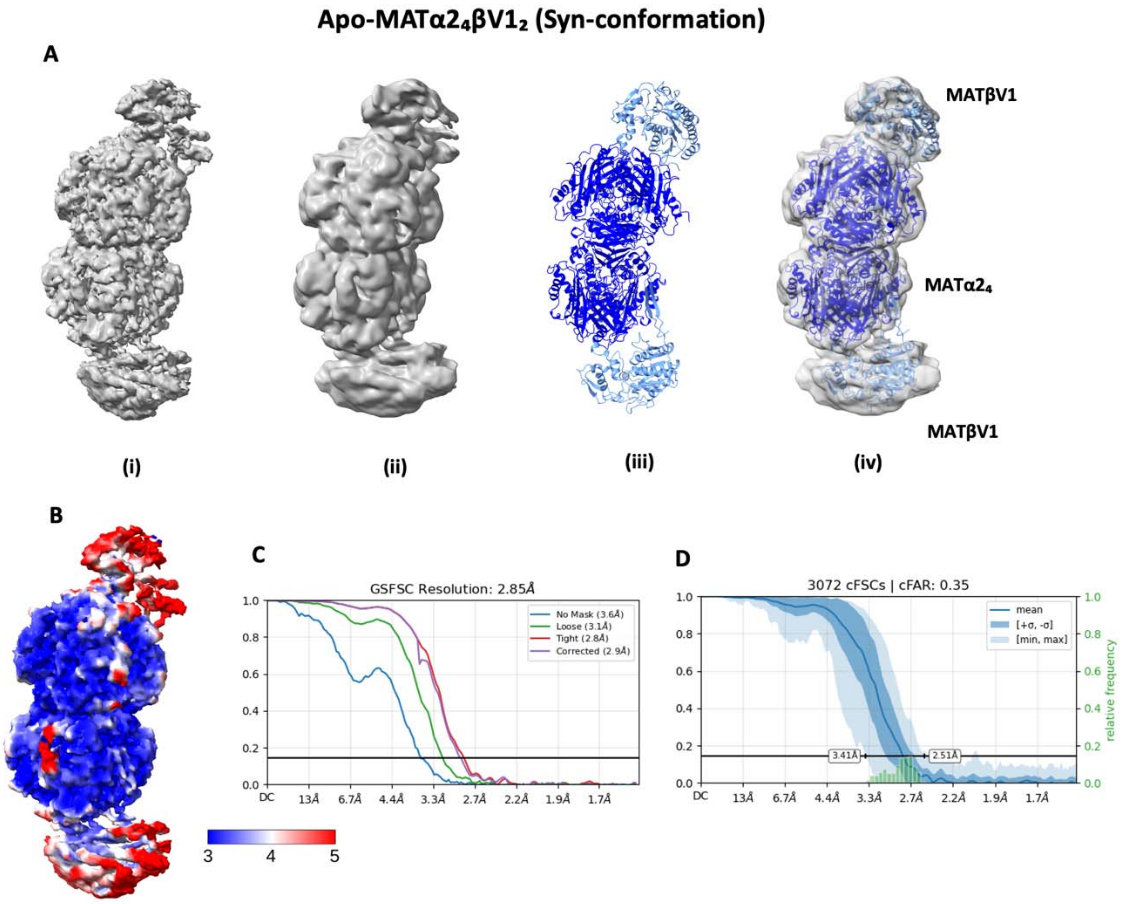


**Figure S19. Model building, FSC curve, cFAR plot, viewing direction distribution, and local resolution analysis for $MAT\alpha2_4\beta V1_2$ in the syn-conformation state. (A)** CryoEM analysis of the $MAT2_4V1_2$ complex in the syn-conformation state: (i) as-obtained cryoEM map; (ii) Gaussian-filtered map with a B-factor of 200; (iii) $MAT2_4V1_2$ model showing $MATV1_2$ in syn conformation; (iv) rigid-body fitting of $MAT2_4V1_2$ complex into the Gaussian-filtered map. (**B**) Local resolution map showing ~3.0 Å resolution in the $MAT2_4$ tetrameric core and ~6Å resolution for the MATV1 subunit near the ends. **(C)** Gold-standard Fourier Shell Correlation (GSFSC) curve for the final reconstruction, indicating a global resolution of 2.85Å at the 0.143 FSC cut-off. **(D)** Conical Fourier Amplitude Ratio (cFAR) plot showing the directional distribution of signal within particles.

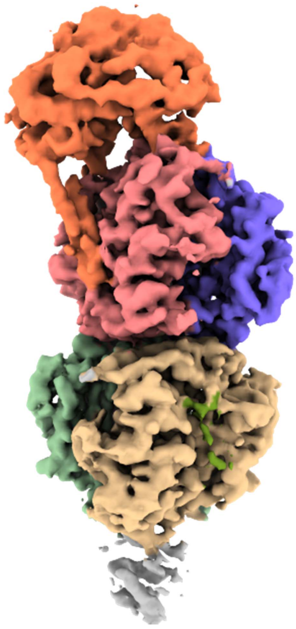

**Supplementary Movie 1. Component 0 of MAT$\alpha 2_4 \beta$V$1_2$ complex (Wobbly) 3DVA; Front view**

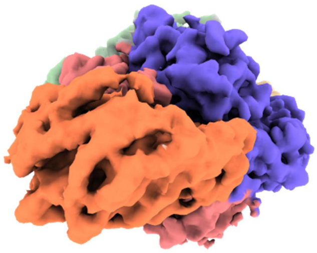

**Supplementary Movie 2. Component 0 of MAT$\alpha 2_4 \beta$V$1_2$ complex (Wobbly) 3DVA; Northern end (view, turn x 90).**

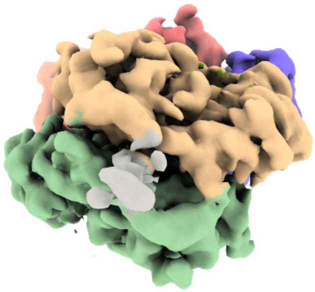

**Supplementary Movie 3. Component 0 of MAT$\alpha 2_4 \beta$V$1_2$ complex (Wobbly) 3DVA; Southern end (view, turn x 270).**

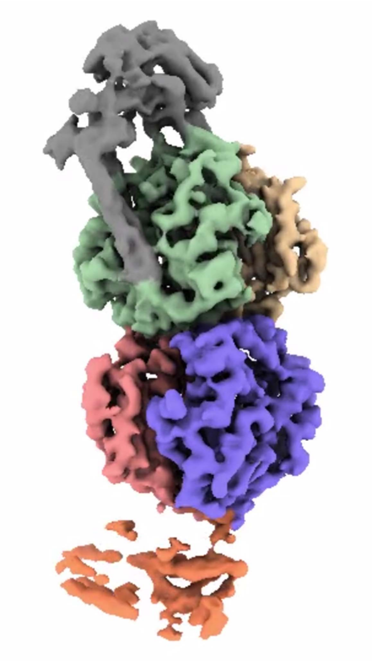

**Supplementary Movie 4. Component 2 of MATα$2_4$βV$1_2$ complex (Syn) 3DVA; Front view**

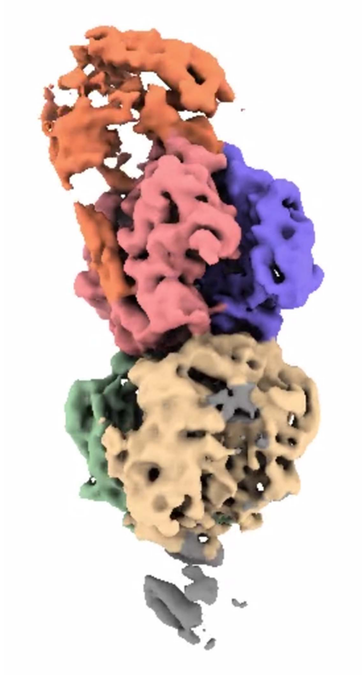

**Supplementary Movie 5. Component 2 of MATα$2_4$βV$1_2$ complex (Anti) 3DVA; Front view**

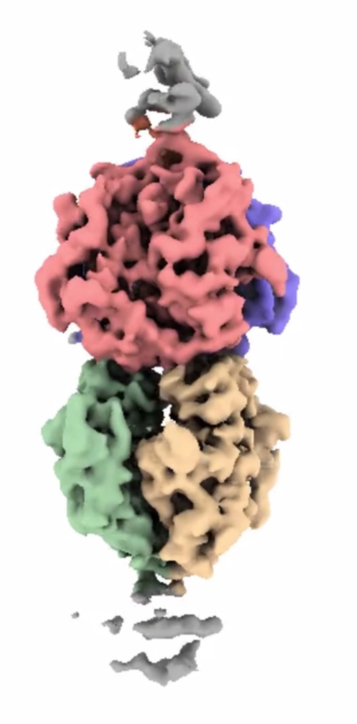

**Supplementary Movie 6. Component 0 of MAT$\alpha 2_4 \beta V2_2$ complex (Wobbly) 3DVA; Front view**

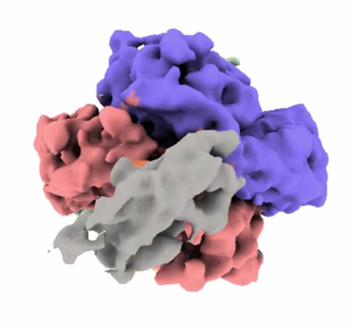

**Supplementary Movie 7. Component 0 of MAT$\alpha 2_4 \beta V2_2$ complex (Wobbly) 3DVA; Northern end (view, turn x 90).**

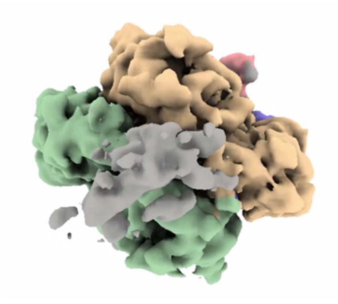

**Supplementary Movie 8. Component 0 of MAT$\alpha 2_4 \beta V2_2$ complex (Wobbly) 3DVA; Southern end (view, turn x 270).**

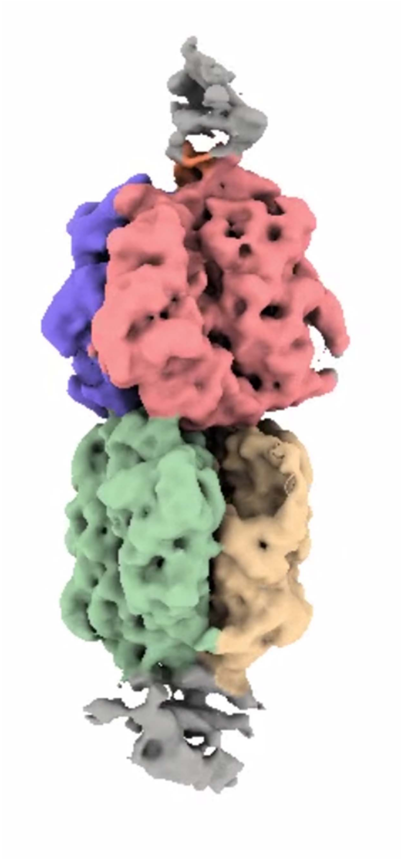

**Supplementary Movie 9. Component 0 of the MATβV2 complex (Wobbly) 3DVA, front view.** The animation illustrates coordinated wobbling motions, highlighting rotational dynamics of the MATV2 subunits and the associated transition of gating loops in all protomers between open and closed conformations.

## References

Murray, B., Antonyuk, S. V., Marina, A., Van Liempd, S. M., Lu, S. C., Mato, J. M., Hasnain, S. S. & Rojas, A. L. (2014). *IUCrJ* **1**, 240–249.

Meng, E. C., Goddard, T. D., Pettersen, E. F., Couch, G. S., Pearson, Z. J., Morris, J. H. & Ferrin, T. E. (2023). *Protein Science* **32**, e4792.

Panmanee, J., Antonyuk, S. V. & Hasnain, S. S. (2020). *Acta Crystallogr D Struct Biol* **76**, 594–607.

Punjani, A., Rubinstein, J. L., Fleet, D. J. & Brubaker, M. A. (2017). *Nat Methods* **14**, 290–296.

Rohou, A. & Grigorieff, N. (2015). *Journal of Structural Biology* **192**, 216–221.

Scheres, S. H. W. (2012). *Journal of Structural Biology* **180**, 519–530.

Wagner, T., Merino, F., Stabrin, M., Moriya, T., Antoni, C., Apelbaum, A., Hagel, P., Sitsel, O., Raisch, T., Prumbaum, D., Quentin, D., Roderer, D., Tacke, S., Siebolds, B., Schubert, E., Shaikh, T. R., Lill, P., Gatsogiannis, C. & Raunser, S. (2019). *Commun Biol* **2**, 218.
